# Physiological models of the lateral superior olive

**DOI:** 10.1371/journal.pcbi.1005903

**Published:** 2017-12-27

**Authors:** Go Ashida, Daniel J. Tollin, Jutta Kretzberg

**Affiliations:** 1 Cluster of Excellence "Hearing4all", Department of Neuroscience, University of Oldenburg, Oldenburg, Germany; 2 Department of Physiology and Biophysics, University of Colorado School of Medicine, Aurora, Colorado, United States of America; University of California at Berkeley, UNITED STATES

## Abstract

In computational biology, modeling is a fundamental tool for formulating, analyzing and predicting complex phenomena. Most neuron models, however, are designed to reproduce certain small sets of empirical data. Hence their outcome is usually not compatible or comparable with other models or datasets, making it unclear how widely applicable such models are. In this study, we investigate these aspects of modeling, namely credibility and generalizability, with a specific focus on auditory neurons involved in the localization of sound sources. The primary cues for binaural sound localization are comprised of interaural time and level differences (ITD/ILD), which are the timing and intensity differences of the sound waves arriving at the two ears. The lateral superior olive (LSO) in the auditory brainstem is one of the locations where such acoustic information is first computed. An LSO neuron receives temporally structured excitatory and inhibitory synaptic inputs that are driven by ipsi- and contralateral sound stimuli, respectively, and changes its spike rate according to binaural acoustic differences. Here we examine seven contemporary models of LSO neurons with different levels of biophysical complexity, from predominantly functional ones (‘shot-noise’ models) to those with more detailed physiological components (variations of integrate-and-fire and Hodgkin-Huxley-type). These models, calibrated to reproduce known monaural and binaural characteristics of LSO, generate largely similar results to each other in simulating ITD and ILD coding. Our comparisons of physiological detail, computational efficiency, predictive performances, and further expandability of the models demonstrate (1) that the simplistic, functional LSO models are suitable for applications where low computational costs and mathematical transparency are needed, (2) that more complex models with detailed membrane potential dynamics are necessary for simulation studies where sub-neuronal nonlinear processes play important roles, and (3) that, for general purposes, intermediate models might be a reasonable compromise between simplicity and biological plausibility.

## Author summary

Computational models help our understanding of complex biological systems, by identifying their key elements and revealing their operational principles. Close comparisons between model predictions and empirical observations ensure our confidence in a model as a building block for further applications. Most current neuronal models, however, are constructed to replicate only a small specific set of experimental data. Thus, it is usually unclear how these models can be generalized to different datasets and how they compare with each other. In this paper, seven neuronal models are examined that are designed to reproduce known physiological characteristics of auditory neurons involved in the detection of sound source location. Despite their different levels of complexity, the models generate largely similar results when their parameters are tuned with common criteria. Comparisons show that simple models are computationally more efficient and theoretically transparent, and therefore suitable for rigorous mathematical analyses and engineering applications including real-time simulations. In contrast, complex models are necessary for investigating the relationship between underlying biophysical processes and sub- and suprathreshold spiking properties, although they have a large number of unconstrained, unverified parameters. Having identified their advantages and drawbacks, these auditory neuron models may readily be used for future studies and applications.

## Introduction

### Concepts of scientific modeling

"All models are wrong but some are useful" [1]. A scientific model can never be ‘true’ or ‘perfect’, since it is, at best, a close approximation of reality. Hence the outcome of a model should not be judged with a simple dichotomy of correct or incorrect, but instead with a graded scale of credibility [2]. Although any scientific theory must be falsifiable [3], it is not falsification itself but the careful scrutiny of the difference between theoretical predictions and empirical data that actually advances our understanding of the modeled system [4,5].

Conventionally, scientific modeling of a complex system is characterized by several guiding principles [4,6,7]: First, a model provides a comprehensive description of the system; second, a model helps in identifying key factors of the system and improves our understanding of its operational rules; third, a model simulates and predicts the outcome of the system; fourth, the outcome of a model simulation confirms or disproves the current hypothesis about the system. In addition to these characteristics, simulations with a well-established model can complement empirical studies. Namely, a theoretical model can guide future experimental research by producing testable predictions [8,9]. Furthermore, components of a model can be easily manipulated in a way that may not be possible with a real system for technical, ethical, or cost-related reasons [4,10]; such efforts include exploratory studies of medical interventions [5]. In order for a model to be credible, it needs to be validated with empirical observations. Validations that are done both with the fundamental (low-level) structure of a model and with its emerging (high-level) outcome ensure our confidence in the predictions of the model [2].

### Lateral superior olive and acoustic information processing

**Fig 1:**
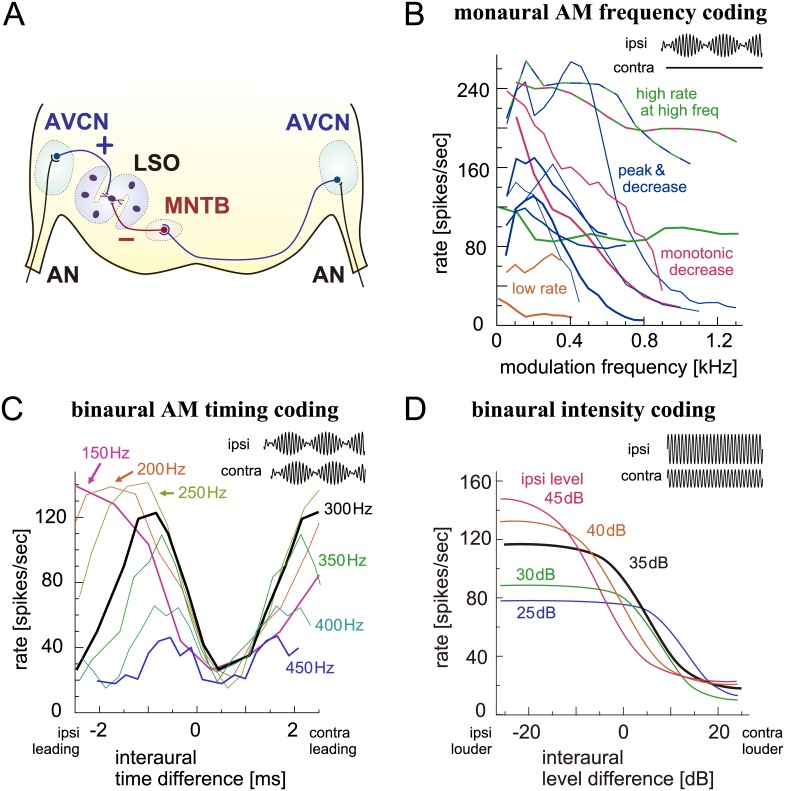
Recorded responses of cat LSO neurons. **A**: Schematic drawing of the LSO circuit. AN: auditory nerve; AVCN: anteroventral cochlear nucleus; MNTB: medial nucleus of the trapezoid body; LSO: lateral superior olive. Excitatory inputs are shown in black and blue, while inhibitory inputs are indicated by red. **B**: Spike rates of LSO neurons in response to monaural AM tones with varied modulation frequencies. Different lines are used for different units; different colors correspond to different response types. Figure taken from [40]; original data collected by Joris and Yin [34]. **C**: Spike rates of an LSO neuron in response to binaural AM tones with varied ITDs. Different lines correspond to different modulation frequencies. Figure taken from [40]; original data collected by Joris and Yin [34]. **D**: Spike rates of LSO a neuron in response to binaural unmodulated tones with varied ILDs. Different colors correspond to different ipsilateral sound levels. Adapted and redrawn from Fig 1A of Tsai et al. [31] with permission.

Here we explore these aspects of computational models with a specific focus on a neuronal circuit that is known to be crucial for sound localization, the auditory function to determine the location of the source of acoustic signals. The two primary cues for sound localization are the timing and intensity differences of the sound arriving at the two ears [11], called interaural time and level differences (ITD/ILD), respectively. Many vertebrates have specialized neuronal circuits in the brainstem for detecting ITDs and ILDs. In mammals, including humans, the lateral superior olive (LSO) is one of such locations where binaural neurons receive inputs originating from the two ears and encode relevant information for sound localization [12]. The principal neuron of the LSO receives excitatory inputs from the spherical bushy cells of the anteroventral cochlear nucleus (AVCN: Fig 1A) [13–21], whose spiking patterns encode timing and intensity information of sounds arriving at the ipsilateral ear. The LSO neuron also receives inhibitory synaptic inputs from neurons in the ipsilateral medial nucleus of the trapezoid body (MNTB: Fig 1A)[17,22–26], which are excited by globular bushy cells in the contralateral AVCN [19,22,24,27–29]. Because of this excitatory-inhibitory interaction, LSO neurons typically show sensitivity to ILDs [30,31]. Namely, the spike rate of an LSO neuron becomes high when the sound source is located in space towards the ipsilateral ear and low when the source is located towards the contralateral ear [32,33]. In addition to this intensity coding, LSO neurons are also sensitive to the timing of sound stimuli; the output spike rate of LSO varies according to the modulation frequency of monaural AM sounds [34] as well as to the ITD of the fine structure [35,36] and envelope of binaural amplitude-modulated (AM) sounds [37–39].

Examples of such timing and intensity coding in the LSO are shown in Fig 1. Although responses of LSO neurons to monaural AM sounds show relatively large variabilities, a majority of neurons presented low- or band-pass characteristics frequently with a mild peak at 100–400 Hz (Fig 1B). This response property can be explained by monaural coincidence detection of excitatory inputs and its variability originates from unit-to-unit differences of biophysical parameters of coincidence detection [40]. The spike rate of the LSO neuron varies periodically with the envelope ITD of AM sounds, with its period being the reciprocal to the modulation frequency (Fig 1C). ITD-tuning curves of LSO neurons to binaural AM sounds at different modulation frequencies are usually aligned at or near their troughs, which is a signature of ‘anti-coincidence detection’ of excitatory and inhibitory synaptic inputs [36,38,40]. The classical ILD-tuning curve of the LSO neuron shows a monotonic sigmoidal decrease of spike rates according to ILDs (Fig 1D). The peak and trough rates as well as the location of the mid-point of the ITD-tuning curve generally depend on the overall input level [31].

### Modeling studies of LSO

A large variety of models have been used to study the functions of LSO. Previous neuronal models of LSO ranged from abstract ones that dealt with the input-output statistics of LSO using point processes [41–44], to a detailed multi-compartment model that incorporated neuronal morphology and spatial distribution of ion channels [45]. Between these two ends of the spectrum, single-compartment (point neuron) models with various internal dynamics have been created, such as simple comparison [46] or temporal summation [47] of excitatory and inhibitory inputs, an electric circuit model of resonating membrane potentials [48], a leaky integrate-and-fire model with standard configurations [49], with synaptic plasticity [50] or with afterhyperpolarization [51], and a Hodgkin-Huxley (HH)-type conductance-based model with several types of ion channels [52,53]; see [54] for a more detailed review of earlier modeling approaches. Recently, LSO models (either abstract or physiology-based) have been incorporated with larger scale simulations, for example, to study psychophysical outcomes [55,56], to develop bio-inspired neural networks of sound localization [57], and to evaluate binaural hearing of cochlear implant users [58,59].

In light of such applications, ‘reproducibility’, ‘credibility’, and ‘generalizability’ comprise fundamental principles of computational models. Reproducibility refers to the ability of the model to (re-)generate sufficiently similar (if not identical) results to the original implementation. Since the importance of reproducible models has been extensively discussed recently (see, e.g., [60,61] and references therein), we do not investigate this issue in the present paper. Credibility refers to the ability of the model to reliably simulate empirical observations. Only with sufficient credibility can a model be reliably used as a building block to construct higher level models [4]. To ensure the credibility of a model, simulated outcome of the model must be validated against corresponding experimental data (see, e.g., [62–64] for the concept of model validation in various scientific fields). Generalizability refers to the applicability of the model to a wide range of contexts including ones that the model was not primarily designed for. In principle, simplistic models with a small number of components are less flexible and less expandable than complex models, resulting in lower generalizability. In the field of neuro- and sensory science, however, most models are tuned to simulate specific sets of experiments. It is therefore normally unclear how a model may or may not reproduce empirical results that are beyond the initial scope of the modeling approach [5,65–67]. The problem of credibility and generalizability also applies to modern LSO modeling; different LSO models are rarely compared with each other, and thus potential users who want to incorporate an LSO model into their simulation framework usually have little or no clue which model to use.

### Models compared in this study

In this study, we introduce, (re-)examine and compare several types of single-compartment LSO neuron models. The complexities of the models, spanning from functional ones that simply compare their excitatory and inhibitory inputs to biophysically detailed conductance-based models whose membrane potential dynamics are determined by nonlinear kinetics of ion channels. Our selection largely covers the spectrum of physiological point neuron models. More specifically, we examine seven LSO models of different levels of complexity: three shot-noise models (coincidence counting model, exponential and alpha Stein models) that simply simulate the excitatory-inhibitory interaction in LSO, and four conductance-based models that describe the membrane potential dynamics of LSO neurons. The conductance-based models can be further subdivided into two classes: integrate-and-fire (IF)-type (passive and active IF models) with explicit threshold parameters, and HH-type (original and adjusted Wang-Colburn models) whose thresholds are determined by the interaction of voltage-dependent conductances.

Among these seven models, the coincidence counting model [40], exponential Stein model [47,54], passive IF model [49–51], and the original Wang-Colburn model [53] were already used in previous modeling studies to simulate monaural or binaural computation of LSO. The alpha Stein and adjusted Wang-Colburn models are modifications of their original counterparts with better biological plausibility or predictive credibility. The active IF model, which is an enhanced version of the standard (passive) IF model with an additional nonlinear conductance, had first been introduced to replicate the activity of auditory coincidence detectors [68], and has been revised here to fit the response properties of LSO.

The parameters of the selected models were tuned to reproduce known *in vivo* and *in vitro* recording results including the monaural and binaural tunings of LSO neurons (as shown in Fig 1), which we considered to be the most representative response properties of LSO (see Discussion). For conductance-based models, sub- and suprathreshold responses of their membrane potentials are also tuned with available physiological data. Since not all models were constructed to replicate all these response properties, calibrating the models at this stage is already part of the generalization process of modeling. After fitting the models, additional model responses and computational performances are compared to further characterize the models. Construction, parameter selection and justification of each model, as well as the response characteristics we examined, are fully described in Materials and Methods with corresponding references.

### Goals of this study

The main goals of this study are to provide several different types of LSO models that are confirmed to be capable of reproducing a pre-defined set of empirical data, and to reveal the fundamental characteristics of these models, so that they can readily be used for future applications. Envisioned usage scenarios range from fundamental biophysical studies to biomedical and engineering applications. Example of fundamental studies include investigating the roles of various ion channels and nonlinear dynamics that determine the excitatory-inhibitory interaction of LSO (e.g., [45,51,53]) and mathematically formulating the input-output relationship of auditory coincidence detectors (e.g., [69,70]). Simulating the binaural hearing of normal listeners [55] and cochlear implant users [58,59,71] is part of possible biomedical applications, while constructions of bio-inspired binaural neuronal networks for mobile robots [72] and cell phone noise reduction [73] are engineering applications that require real-time computation. Since there is a general trade-off between simplicity and biological plausibility [9], selecting the most suitable model should depend on the purposes of a specific application. The present study thus aims to reveal the advantages and disadvantages of each LSO model to help future users select an appropriate model for their envisaged use.

In the following sections, we examine each LSO model in detail. Based on our comparison results of the seven LSO models that are tuned (generalized) with the common criteria, we conclude that the simplistic shot-noise models are suitable for applications where computational efficiency or theoretical transparency is desired, while the more complex conductance-based models are generally required for investigating the underlying sub-neuronal mechanisms of binaural computation. Within the conductance-based models, either HH-type models or IF-type models should be selected by the user, depending on the required details of discharging mechanisms and morphological expandability [74].

## Results

### Comparison of LSO models: General setup

In this study, we compare the seven physiological models of LSO that were briefly introduced in the last section. The full definitions of the models and the detailed criteria for selecting their parameters are provided in Materials and Methods. In this section, we briefly describe the underlying ideas of calibration and evaluation of the models. In next sections, systematic examinations of each model will follow.

**Fig 2:**
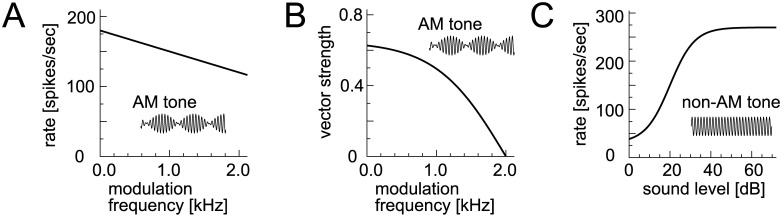
Modeled input rates. **A**: Modeled modulation-frequency dependence of spike rates of bushy cells and MNTB neurons driven by AM tones. **B**: Modeled modulation-frequency dependence of phase-locking of bushy cells and MNTB neurons driven by AM tones. **C**: Modeled level-dependence of spike rates of bushy cells and MNTB neurons driven by unmodulated tones. See "Common input" in Materials and methods for the equations.

Following our previous work [40], the input stage of the modeling framework consists of 20 excitatory fibers and 8 inhibitory fibers, which correspond to bushy cells in the AVCN and principal neurons in the MNTB, respectively (Fig 1A). The spiking patterns of these input fibers are modeled with an inhomogeneous Poisson process to simulate empirical spike rates and degree of phase-locking, both of which varied with the modulation frequency and the sound level (Fig 2; see Materials and methods for relevant references). To enable direct comparisons across LSO models, the same set of simulated inputs is fed to all the model neurons; namely, excitatory and inhibitory presynaptic spikes generated by Poisson processes with the identical random seed are commonly given to each model as its input.

Each LSO neuron model has its own set of parameters, some of which are experimentally constrained (i.e., corresponding empirical data exist) and others are not. We used data from cats, gerbils, guinea pigs, rats, and mice to calibrate and justify the parameters of each LSO model (see specific section for each model in Materials and methods). Experimental measurements, however, are generally subject to random noise, trial-to-trial variability, and unit-to-unit variability. Therefore, empirical data for any specific parameter are usually reported as a range, but not as a single value. Wherever possible, we tried selecting parameters from empirically measured ranges. In conductance-based models, we first fit the parameters for subthreshold responses, and next tuned the remaining parameters for spiking output.

**Fig 3:**
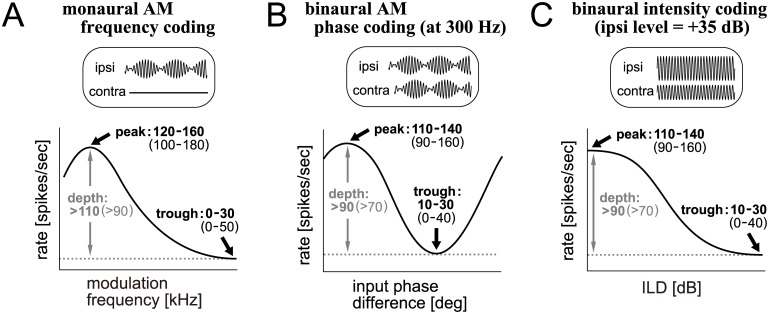
Targeted output rates. **A**: Targeted modulation-frequency dependence response of LSO models driven by monaural AM tones. **B**: Targeted phase-difference-dependent response of LSO models driven by binaural AM tones. **C**: Targeted ILD-dependent response of LSO neurons driven by binaural unmodulated tones. In A-C, ‘targeted’ values are shown in bold, and ‘accepted’ values are in non-bold (see ‘Output measures’ in Materials and methods for their definitions and relevant descriptions).

To facilitate comparisons across models, we selected the parameters so that the model output resembled empirical results. The output measures we used are fully described in Materials and Methods with relevant references. In brief, we calibrated the model with the monaural AM frequency tuning curve for the modulation frequency between 50 and 1200 Hz (Fig 3A), the binaural AM phase tuning curve at the modulation frequency of 300 Hz (Fig 3B), and the binaural level tuning curve at the ipsilateral level of +35 dB (Fig 3C). For each of these tuning curves, the peak rate, trough rate, and the modulation depth (defined as peak rate minus trough rate) were determined. For each of these three rates in each of the three tuning curves, we defined a ‘target’ range (bold numbers in Fig 3). Model parameters were selected such that the resulting spike rates fell within these target ranges. If we did not find a combination of parameters that satisfies all the target ranges, we loosened the criteria by adopting the ‘accepted’ ranges (non-bold numbers in Fig 3), and re-selected the model parameters. In searching parameters, we did not use fully automated methods such as genetic algorithms, but chose one of the proper parameter sets in a semi-manual way by dividing the parameter space into grids (see Discussion). After generalizing the models with the common tuning criteria, we also calculated the binaural tuning curves at different modulation frequencies (150 and 450 Hz) or at different ipsilateral sound levels (+25 to +45 dB) to further characterize the model.

### Coincidence counting model

**Fig 4:**
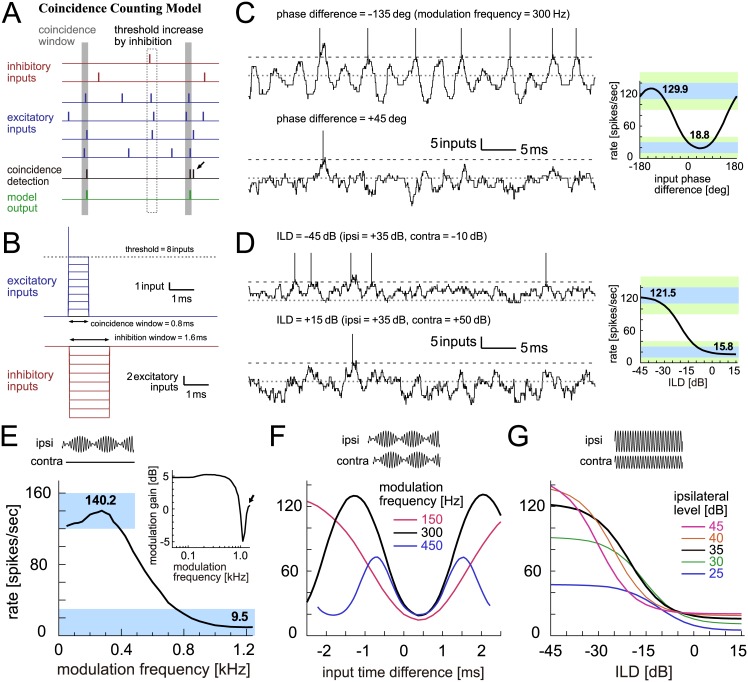
Coincidence counting model of LSO. **A**: Schematic drawing of the coincidence counting model. Each vertical bar corresponds to a spike (red: inhibitory inputs; blue: excitatory inputs; black: detected coincidence; green: spike output of the model). An input coincidence is counted when the number of inputs in the coincidence window W_ex_ (shaded vertical rectangle) reaches or exceeds the threshold θ (θ = 3 in this particular example). The small black arrow indicates an output spike rejected by the refractory period T. Each inhibitory input removes H excitatory input in the inhibition window W_inh_ (dotted vertical rectangle). **B**: Modeled excitatory and inhibitory synaptic inputs. The duration of an excitatory input is described as the coincidence window of W_ex_, whereas the duration of an inhibitory input is modeled as the inhibition window of W_inh_. The effect of inhibitory inputs is modeled as twice as that of an excitatory input (i.e., H = 2). Actual parameters used are summarized in Materials and Methods. **C**: (Left) Modeled traces of the coincidence counts driven by binaural AM tones with two different input phase differences. (Right) Output rates of the model in response to binaural AM tones with varied input phase differences. Bold numbers show the peak and trough rates. **D**: (Left) Modeled traces of the coincidence counts driven by binaural unmodulated tones with two different ILDs. (Right) Output rates of the model in response to binaural unmodulated tones with varied ILDs. Bold numbers show the rates at -45 dB and +15 dB. In panels C (Left) and D (Left), horizontal dotted gray lines and broken black lines indicate the zero input level and the threshold, respectively. At each threshold crossing, a vertical line was manually added to show the generation of an output spike. **E**: Monaural AM-tuning curve (rate-MTF) of the coincidence counting model. Bold numbers show the peak rate and the rate at 1200 Hz. (Inset) Monaural phase-locking (synch-MTF) of the model. Blue rectangular shading in C-E indicates the targeted ranges, while green shading in C-D shows the accepted ranges. **F**: Binaural AM phase-tuning curves of the model at three modulation frequencies. **G**: Binaural ILD-tuning curves of the model at five ipsilateral sound levels.

The coincidence counting model was introduced to explain how ‘anti-coincidence’ of excitatory and inhibitory synaptic inputs affects the monaural and binaural coding in the LSO [40]. The model simply counts the number of synchronized excitatory inputs arriving within the coincidence window and generates a spike if this number reaches or exceeds the threshold (Fig 4A). Effects of inhibition are modeled as a subtraction of excitatory inputs (or equivalently as an increase of the threshold) within the inhibition time window. Excitation and inhibition have different amplitudes and time scales (Fig 4B), which were selected to fit the target output rates.

Sample traces of the model are shown in Fig 4C and 4D; since each synaptic input was modeled as a rectangle with an abrupt onset and offset (Fig 4B), simulated subthreshold model traces were generally jaggy (Fig 4C, left; Fig 4D, left). For phase-locked inputs driven by AM sounds, the intensity of summed synaptic inputs changed periodically at the modulation frequency of the sound. When the excitatory and inhibitory inputs were out of phase, the subthreshold response (virtual membrane potential) of the model showed large oscillations and the resulting output rate becomes high (Fig 4C, top left). When the excitation and inhibition arrived in phase, they canceled each other, resulting in a low output rate (Fig 4C, bottom left). Because of this excitation-inhibition interaction, the output rate of the model neuron changed according to the phase difference of simulated excitatory and inhibitory synaptic inputs (Fig 4C, right). For non-phase-locked inputs (corresponding to non-modulating sound stimuli as in Fig 1D), the output rate of the model neuron depended on the relative intensities of excitatory and inhibitory inputs (Fig 4D, right). When the sound level was higher at the ipsilateral ear than at the contralateral ear, excitatory inputs strongly drove the model neuron, leading to a high output spike rate (Fig 4D, top left). As the sound level at the contralateral ear increased, intensity of inhibitory inputs became stronger, making the virtual membrane potential stay away from the spike threshold (Fig 4D, bottom left).

The simulated monaural tuning curve (rate modulate transfer function: rate-MTF) for the coincidence counting model showed a mild peak at 200–300 Hz (Fig 4E), corresponding to the ‘peak & decrease’ type of empirical tuning curves (Fig 1B). This peak was explained by monaural coincidence detection of excitatory inputs [40]. The degree of phase-locking, measured by modulation gain (synchrony modulation transfer function: synch-MTF), showed similar patterns to experimental data [34], with a mild peak found at 200–500 Hz (Fig 4E, inset). However, the synch-MTF showed a rebound at above 1 kHz (Fig 4E, inset, arrow), which was not seen in previous recordings (e.g., [34]).

Simulated binaural phase-tuning curves at 150, 300 and 450 Hz (Fig 4F) all resembled empirical data (Fig 1C), with troughs aligned at a positive time difference. Simulated binaural level-tuning curves at different ipsilateral levels (Fig 4G) were also similar to empirical data (Fig 1D), with level-dependent peak rates and midpoint positions. In the following sections, we compare these monaural and binaural tuning properties between LSO models.

### Stein models

The Stein model is named after Richard B. Stein [75,76], who introduced the model to theoretically investigate the variability of neuronal spiking activity. This model was later adopted for the study of the binaural function of LSO [47]. The type of model is also called the ‘shot-noise’ model [54], but we use this term for a wider category that includes both the coincidence counting model and the Stein models. In this paper, we compare two types of Stein models that are distinguished by the function used for synaptic inputs; namely, the conventional version named the ‘exponential Stein model’ with exponentially decaying functions, and the revised version, the ‘alpha Stein model’, using alpha functions. These Stein models have more flexibility than the coincidence counting model, since they have decaying synaptic inputs and thus allow for non-integer thresholds. The internal state of the model reflecting the decaying synaptic inputs is here called the ‘virtual membrane potential’. The term ‘virtual’ is used to indicate the fact that a shot-noise model does not have an explicit membrane potential (in mV) but instead counts the number of input spikes as an analog of the membrane response.

#### Exponential Stein model

**Fig 5:**
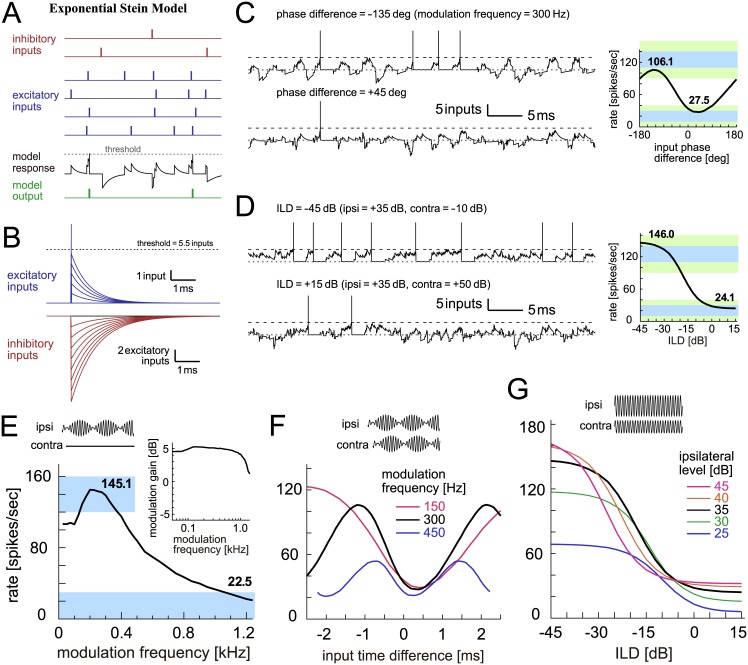
Exponential Stein model of LSO. **A**: Schematic drawing of the exponential Stein model. Each vertical bar corresponds to a spike (red: inhibitory inputs; blue: excitatory inputs; black: internal state (i.e., virtual membrane potential) of the model; green: spike output of the model). Synaptic inputs are modeled as exponentially decaying functions and linearly summed to produce the internal state of the model. An output spike is generated when the sum of inputs reaches or exceeds the threshold. The internal state is reset to and fixed at zero during the refractory period after each spike. **B**: Excitatory and inhibitory synaptic inputs modeled as exponentially decaying functions with different amplitudes and time constants. **C**: (Left) Traces of the internal state of the model driven by binaural AM tones with two different input phase differences. (Right) Output rates of the model in response to binaural AM tones with varied input phase differences. Bold numbers show the peak and trough rates. **D**: (Left) Traces of the internal state driven by binaural unmodulated tones with two different ILDs. (Right) Output rates of the model in response to binaural unmodulated tones with varied ILDs. Bold numbers show the rates at -45 dB and +15 dB. In panels C (Left) and D (Left), horizontal dotted gray lines and broken black lines indicate the zero input level and the threshold, respectively. At each threshold crossing, a vertical line was manually added to show the generation of an output spike. **E**: Monaural AM-tuning curve (rate-MTF) of the exponential Stein model. Bold numbers show the peak rate and the rate at 1200 Hz. (Inset) Monaural phase-locking (synch-MTF) of the model. Blue rectangular shading in C-E indicates the targeted ranges, while green shading in C-D shows the accepted ranges. **F**: Binaural AM phase-tuning curves of the model at three modulation frequencies. **G**: Binaural ILD-tuning curves of the model at five ipsilateral sound levels.

In the original type of the Stein model, which we here call the ‘exponential Stein model’, each synaptic input is converted into an exponential function (Fig 5A). Excitatory and inhibitory inputs are represented as positive and negative changes in the virtual membrane potential, respectively (Fig 5B). An output spike is generated when the virtual membrane potential reaches or exceeds the threshold. The amplitude and the time constant of exponential curves for unitary synaptic inputs were tuned to fit the output criteria of monaural and binaural responses (Fig 5C, right; Fig 5D, right; and Fig 5E, main panel). It should be noted, however, that the threshold level found in our parameter search (5.5 inputs) was considerably lower than the corresponding empirical data (9.6 ± 2.8 inputs in gerbils [77]) (see Materials and methods for more detail on parameter selection). This discrepancy was primarily due to the unrealistic shape of the modeled synaptic input that is extremely steep at the onset and decays rather slowly afterwards. Due to this sharp onset, the simulated traces are jagged with synaptic inputs (Fig 5C and 5D, left).

Simulated monaural and binaural tuning curves (Fig 5E–5G) were roughly similar to empirical data (Fig 1B–1D). However, some of the tuning properties were different from the coincidence counting model. The rate-MTF (Fig 5E) for the exponential Stein model decreased slightly more slowly with the modulation frequency than for the coincidence counting model. The synch-MTF (Fig 5E, inset) did not decay at the modulation frequencies below 1 kHz. The trough positions of the binaural phase tuning curves did not perfectly align (Fig 5F) because of the temporally asymmetric synaptic inputs. Peak spike rates of the binaural level tuning curve (Fig 5G) tended to be higher than for the coincidence counting model. This is explained by the temporal summation of synaptic inputs: i.e., in the coincidence counting model, the effects of each excitatory synaptic inputs lasts only for the time window of W_ex_ = 0.8 ms (Fig 4B), whereas, in the exponential Stein model, synaptic inputs may sum up for a considerably longer time scale (Fig 5B). These simulation results suggest that the modeled synaptic inputs of the exponential Stein model may have to be revised to achieve a better fit with empirical data, although the general principle of the excitatory-inhibitory interactions is captured by the model [54]. This motivated us to introduce the alpha Stein model (next section).

#### Alpha Stein model

**Fig 6:**
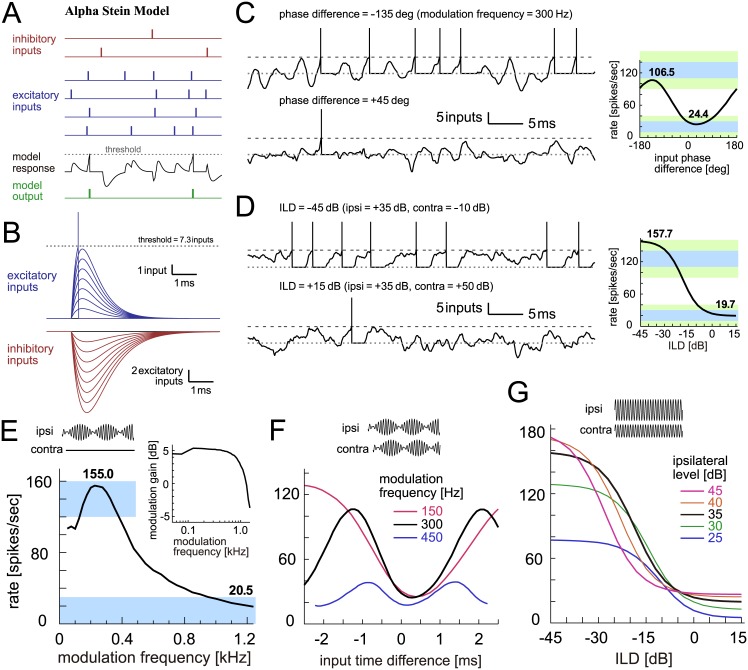
Alpha Stein model of LSO. **A**: Schematic drawing of the alpha Stein model. Each vertical bar corresponds to a spike (red: inhibitory inputs; blue: excitatory inputs; black: internal state (i.e., virtual membrane potential) of the model; green: spike output of the model). Synaptic inputs are modeled as alpha functions and linearly summed to produce the internal state of the model. An output spike is generated when the sum of inputs reaches or exceeds the threshold. The internal state is reset to and fixed at zero during the refractory period after each spike. **B**: Modeled excitatory and inhibitory synaptic inputs. Excitatory and inhibitory synaptic inputs are both converted into alpha functions, but with different amplitudes and time constants. **C**: (Left) Traces of the internal state of the model driven by binaural AM tones with two different input phase differences. (Right) Output rates of the model in response to binaural AM tones with varied input phase differences. Bold numbers show the peak and trough rates. **D**: (Left) Traces of the internal state driven by binaural unmodulated tones with two different ILDs. (Right) Output rates of the model in response to binaural unmodulated tones with varied ILDs. Bold numbers show the rates at -45 dB and +15 dB. In panels C (Left) and D (Left), horizontal dotted gray lines and broken black lines indicate the zero input level and the threshold, respectively. At each threshold crossing, a vertical line was manually added to show the generation of an output spike. **E**: Monaural AM-tuning curve (rate-MTF) of the alpha Stein model. Bold numbers show the peak rate and the rate at 1200 Hz. (Inset) Monaural phase-locking (synch-MTF) of the model. Blue rectangular shading in C-E indicates the targeted ranges, while green shading in C-D shows the accepted ranges. **F**: Binaural AM phase-tuning curves of the model at three modulation frequencies. **G**: Binaural ILD-tuning curves of the model at five ipsilateral sound levels.

**Fig 7:**
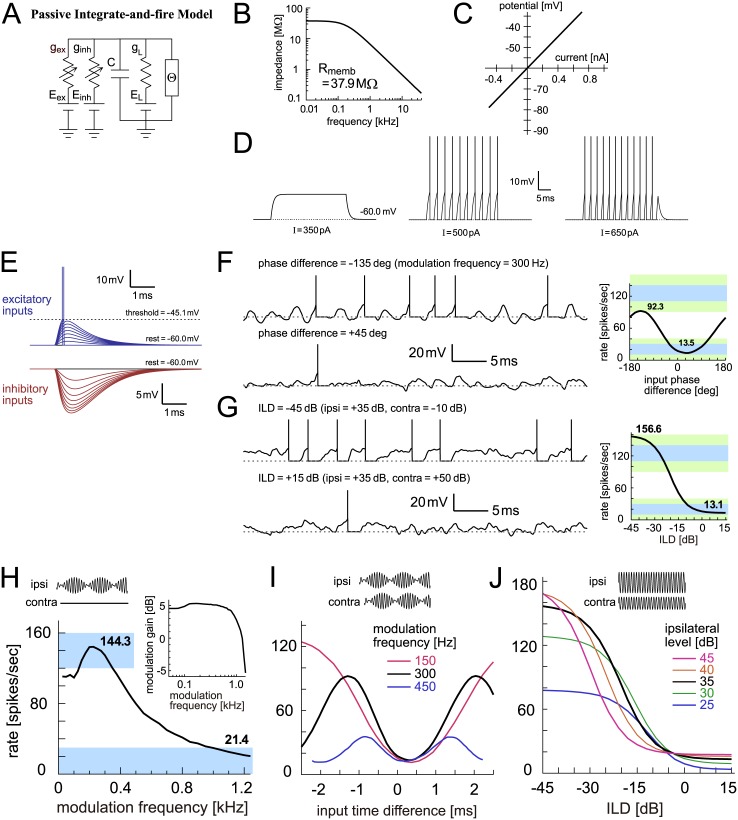
Passive integrate-and-fire model of LSO. **A**: Circuit diagram of the passive IF model. Θ denotes the threshold crossing detector. **B**: Membrane impedance of the model. **C**: Current-potential (I-V) relationship of the model. **D**: Model responses to step current input with three varied sizes. **E**: Membrane responses to modeled excitatory and inhibitory synaptic inputs. **F**: (Left) Modeled membrane potentials driven by binaural AM tones with two different input phase differences. (Right) Output rates of the model in response to binaural AM tones with varied input phase differences. Bold numbers show the peak and trough rates. **G**: (Left) Modeled membrane potential driven by binaural unmodulated tones with two different ILDs. (Right) Output rates of the model in response to binaural unmodulated tones with varied ILDs. Bold numbers show the rates at -45 dB and +15 dB. In panels F (Left) and G (Left), horizontal dotted gray lines indicate the resting potential. In panels D-G, vertical bars were manually added to show the generation of an output spike at each spike crossing. **H**: Monaural AM-tuning curve (rate-MTF) of the passive IF model. Bold numbers show the peak rate and the rate at 1200 Hz. (Inset) Monaural phase-locking (synch-MTF) of the model. Blue rectangular shading in F-H indicates the targeted ranges, while green shading in F-G shows the accepted ranges. **I**: Binaural AM phase-tuning curves of the model at three modulation frequencies. **J**: Binaural ILD-tuning curves of the model at five ipsilateral sound levels.

In the alpha Stein model, unitary synaptic inputs are formulated as an alpha function (Fig 6A and 6B), while the other operations remain unchanged from the exponential Stein model (see Materials and methods). The selected threshold of this model (7.3 inputs) was higher than that of the exponential Stein model (5.5 inputs) and better matched corresponding empirical data (9.6 ± 2.8 inputs [77]). Simulated traces (Fig 6C and 6D, left) were smoother than those of the coincidence counting and exponential Stein models, although the simulated monaural and binaural tuning curves did not greatly differ (Fig 6C–6E). The synch-MTF started to decay at a lower modulation frequency than for the exponential Stein model (Fig 6E, inset). Because the modeled synaptic input has a steeper onset than the decay, there was a slight offset between the trough positions (Fig 6F). Relatively higher peaks for high-intensity inputs (Fig 7G) were found, as similarly seen in the exponential Stein model.

### Integrate-and-fire models

The integrate-and-fire (IF) model and its variations have been widely used in theoretical and computational neuroscience [78–80], including modeling studies of LSO [49–51]. Compared to the shot-noise models examined above, IF-type models can be more directly related to biological membranes by having variables and parameters with clear biophysical meanings, such as the membrane potential, input resistance, and the membrane time constant. Here we examine two IF-type models: the standard leaky (linear) IF model, which we call the ‘passive IF model’, and an enhanced version with a nonlinear subthreshold current, which we call the ‘active IF model‘. The general idea of the active IF model was previously presented in [68], but its membrane properties and spike-associated current were revised to fit known physiological characteristics of LSO. In both models, we first tuned the subthreshold membrane parameters using empirical data, and then selected the threshold to reproduce monaural and binaural responses (see Materials and methods for details).

#### Passive integrate-and-fire model

In the passive IF model, the subthreshold response of the membrane is described as an RC circuit (Fig 7A). Therefore its impedance profile is low-pass (Fig 7B), and its I-V relationship is linear (Fig 7C). In response to step (rectangular) current injections, the model produced either no spikes (Fig 7D, left) or repetitive spikes (Fig 7D, middle and right), depending on the amplitude of the current. Summation of inhibitory synaptic inputs (Fig 7E, bottom) was sublinear because its reversal potential was only about 15 mV below the resting potential (i.e., E_inh_ = -75 mV). In contrast, since the excitatory reversal potential was far above the resting potential (E_ex_ = 0 mV), excitatory inputs summed almost linearly (Fig 7E, top).

Responses to modeled synaptic inputs in the passive-IF model (Fig 7F and 7G, left) resembled those in the alpha Stein model, but slightly less noisy because the low-pass membrane filtered out high-frequency fluctuations. Simulated monaural and binaural tuning curves (Fig 7F and 7G, right; Fig 7H) also had similar shapes. The trough positions of the phase-tuning curves (Fig 7I), however, showed a better alignment than the Stein models, since the low-pass membrane resulted in somewhat more symmetrical shapes in synaptic potentials. The level-tuning curves (Fig 7J) were almost identical to the alpha Stein model, with peaks higher than empirical data (Fig 1D), since the model lacks mechanisms to compress high intensity inputs (see Discussion).

#### Active integrate-and-fire model

**Fig 8:**
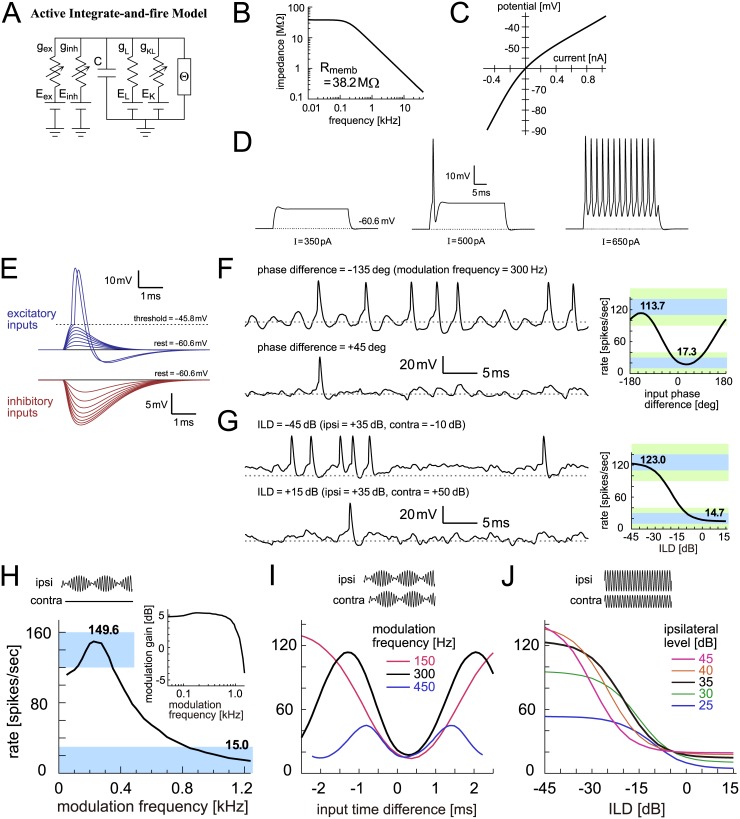
Active integrate-and-fire model of LSO. **A**: Circuit diagram of the active IF model. Θ denotes the threshold crossing detector and spike current generator. **B**: Membrane impedance of the model. **C**: Current-potential (I-V) relationship of the model. **D**: Model responses to step current input with three varied sizes. **E**: Membrane responses to modeled excitatory and inhibitory synaptic inputs. **F**: (Left) Modeled membrane potentials driven by binaural AM tones with two different input phase differences. (Right) Output rates of the model in response to binaural AM tones with varied input phase differences. Bold numbers show the peak and trough rates. **G**: (Left) Modeled membrane potential driven by binaural unmodulated tones with two different ILDs. (Right) Output rates of the model in response to binaural unmodulated tones with varied ILDs. Bold numbers show the rates at -45 dB and +15 dB. In panels F (Left) and G (Left), horizontal dotted gray lines indicate the resting potential. **H**: Monaural AM-tuning curve (rate-MTF) of the active IF model. Bold numbers show the peak rate and the rate at 1200 Hz. (Inset) Monaural phase-locking (synch-MTF) of the model. Blue rectangular shading in F-H indicates the targeted ranges, while green shading in F-G shows the accepted ranges. **I**: Binaural AM phase-tuning curves of the model at three modulation frequencies. **J**: Binaural ILD-tuning curves of the model at five ipsilateral sound levels.

In addition to the membrane capacitance and the linear leak conductance, the active IF model has a non-linear low-voltage-activated potassium (KLVA) conductance (Fig 8A). KLVA channels are commonly found along the auditory pathway and play an important role in determining the temporal response properties of auditory neurons (see [81,82] for reviews). The impedance profile of the membrane was still low-pass (Fig 8B), whereas a much larger amount of KLVA conductance in other auditory coincidence detector models was found to make the membrane weakly band-pass [83,84]. KLVA current is activated near or slightly above the resting potential, resulting in lower membrane impedances at depolarized potentials (Fig 8C) and a phasic (onset) spiking response to a step current of an intermediate amplitude (Fig 8D, middle). The transition from phasic to tonic spiking was seen in slice recordings from rat LSO [85] corresponding to the tonotopic distribution of KLVA conductance [86].

Instead of the simple potential reset, an additional spike-associated current was injected after each threshold crossing (Fig 8E) in our active IF model. This spike-mimicking current was introduced to make the simulated potential traces (Fig 8F and 8G, left) more realistic than other simpler models. Monaural (Fig 8H) and binaural responses (Fig 8F and 8G right) of the active IF model were all within the targeted ranges (Fig 3). The rate-MTF (Fig 8H, main panel), synch-MTF (Fig 8H, inset) and binaural phase-tuning curve (Fig 8I) resembled those of the passive IF model. The binaural level-tuning curve (Fig 8J), however, showed lower peak rates especially with high ipsilateral levels, better replicating the empirical data (Fig 1D). This was achieved by the KLVA conductance, which compressed the accumulated input by making the input resistance low at depolarized membrane potentials (Fig 8C).

### Wang-Colburn models

**Fig 9:**
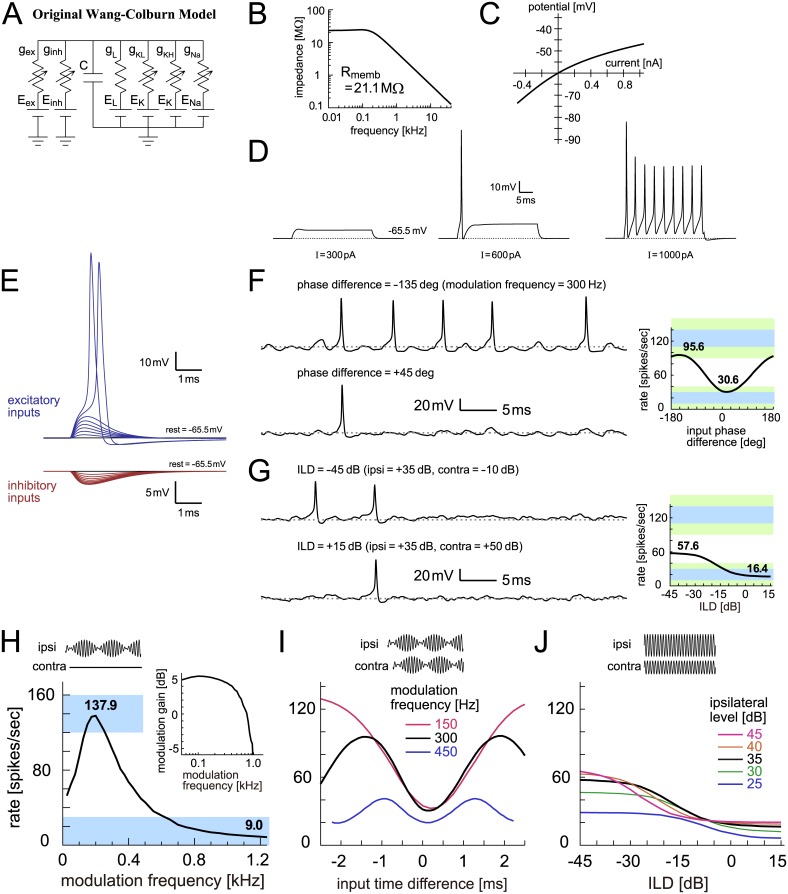
Original Wang-Colburn model of LSO. **A**: Circuit diagram of the original Wang-Colburn model. **B**: Membrane impedance of the model. **C**: Current-potential (I-V) relationship of the model. **D**: Model responses to step current input with three varied sizes. **E**: Membrane responses to modeled excitatory and inhibitory synaptic inputs. **F**: (Left) Modeled membrane potentials driven by binaural AM tones with two different input phase differences. (Right) Outputs rate of the model in response to binaural AM tones with varied input phase differences. Bold numbers show the peak and trough rates. **G**: (Left) Modeled membrane potential driven by binaural unmodulated tones with two different ILDs. (Right) Output rates of the model in response to binaural unmodulated tones with varied ILDs. Bold numbers show the rates at -45 dB and +15 dB. In panels F (Left) and G (Left), horizontal dotted gray lines indicate the resting potential. **H**: Monaural AM-tuning curve (rate-MTF) of the original Wang-Colburn model. Bold numbers show the peak rate and the rate at 1200 Hz. (Inset) Monaural phase-locking (synch-MTF) of the model. Blue rectangular shading in F-H indicates the targeted ranges, while green shading in F-G shows the accepted ranges. **I**: Binaural AM phase-tuning curves of the model at three modulation frequencies. **J**: Binaural ILD-tuning curves of the model at five ipsilateral sound levels.

The Wang-Colburn model [53] is a conductance-based, HH-type model with several nonlinear conductances (Fig 9A). The leak and KLVA currents characterize the subthreshold responses, whereas the high-voltage activated potassium (KHVA) and sodium (Na) currents are responsible for spike initiation and after-spike repolarization. The kinetic equations for the Wang-Colburn model was taken from the Rothman-Manis model [87], which was based on physiological recordings of guinea pigs and has been widely used in computational studies of auditory neuroscience. The model does not have an explicit spike threshold as a parameter, since the spike threshold of a HH-type model is determined by nonlinear interactions of the ionic conductances. Because of these characteristics, the Wang-Colburn model has more plausible biological grounds than other simpler models that we have examined so far. It nevertheless has a number of parameters that are not physiologically well-constrained (i.e., not all parameters were empirically measured, or even measurable). In the present study, we examine two types of the Wang-Colburn models: the original version [53] and an adjusted version to better fit empirical data (see Materials and methods for detailed definitions).

#### Original Wang-Colburn model

In the original Wang-Colburn model, the values for the membrane capacitance and the leak conductance were taken from their prior study using an IF-type model [51], and were considerably larger than the values used for the other models in our present study. The resulting membrane constant of the Wang-Colburn model was 1.0 ms, comparable to the empirical values in gerbils (1.1 ± 0.4 ms: [77]), while the input resistance of 21.1 MΩ was relatively lower than the measured values in the same species (42 ± 21 MΩ: [77]). Because of the large amount of potassium conductances, the impedance was highest at 100–200 Hz, although the overall shape of the impedance curve was nearly low-pass (Fig 9B). The I-V curve showed an outward rectification (Fig 9C). Due to the activation of KLVA and inactivation of Na conductances near the resting potential, the excitability of this model was substantially lower than other models, showing phasic spiking even in response to a large step current (Fig 9D, middle).

Since the resting potential (around -65 mV) was close to the inhibitory reversal potential of this model (E_in_ = -70 mV), simulated inhibitory inputs (Fig 9E, bottom; unitary amplitude = 0.6–0.7 mV) were much smaller than the measured inhibitory inputs (1.6–8.3 mV: [77]). Spike peaks of the original Wang-Colburn model reached +10 to +30 mV (Fig 9E, top; Fig 9F, left; Fig 9G, left), resembling LSO neurons in mice [88] and rats [85], rather than gerbil LSO neurons that rarely showed overshooting [77]. Because of the weak inhibitory inputs, the output rate did not fall below 20 spikes/s, even when inhibitory input arrived in-phase with excitatory inputs (Fig 9F, right). Furthermore, because of the low excitability of the model neuron, the simulated maximum rate was only about 60 spikes/sec when driven by non-phase-locked excitatory inputs (Fig 9G, right).

As reported previously [53], simulated monaural AM tuning curve showed a peak around 200 Hz (Fig 9H). Synch-MTF decreased more rapidly even below 1 kHz (Fig 9H, inset) than other models. The binaural AM phase tuning curves roughly aligned at the troughs (Fig 9I), but the depths were generally shallow. The most notable difference from other models (and from empirical data) was the low output rates in the binaural level tuning curves (Fig 9J). This was due to the large potassium conductance in combination with the strong sodium inactivation, both of which hinder repetitive spiking when driven by continuous inputs, as seen with step currents (Fig 9D, middle).

#### Adjusted Wang-Colburn model

The major discrepancies found between the original Wang-Colburn model and empirical data necessitated a revision of the model. We thus made the following changes to obtain an adjusted version of the Wang-Colburn model. First, we shifted the voltage dependence of the ion channel kinetics to reduce the effects of KLVA activation and Na inactivation. Next, we revised the membrane capacitance, leak and KLVA conductances, and reversal potentials to gain a better fit to corresponding experimental data. Finally, the values of Na and KHVA conductances were selected to achieve our output criteria of monaural and binaural tunings. More detailed descriptions of the model adjustment are provided in Materials and Methods.

**Fig 10:**
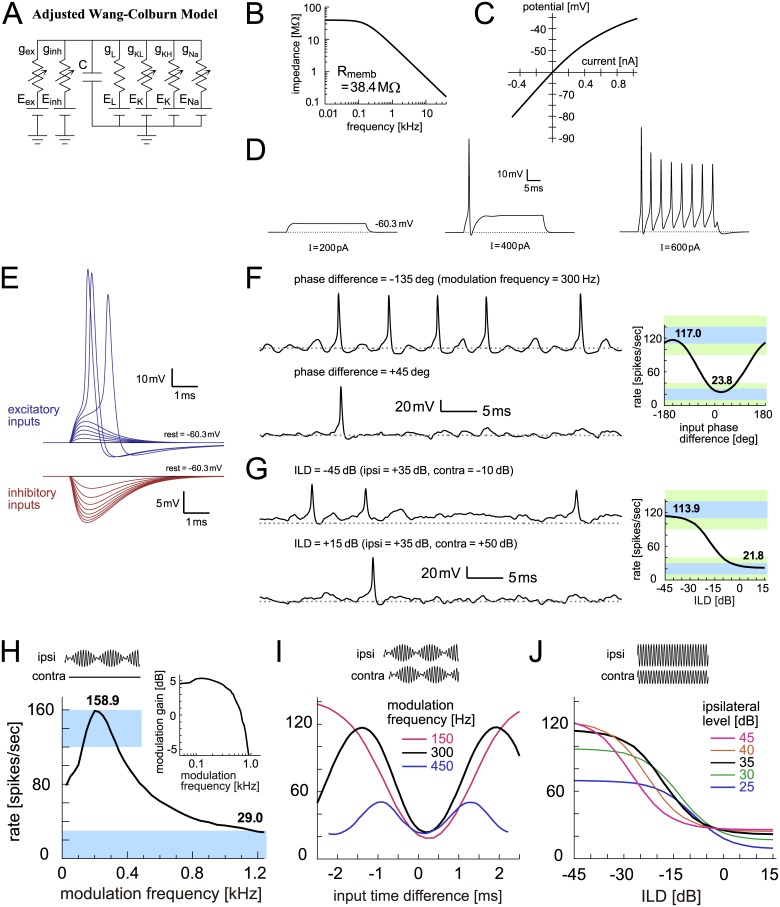
Adjusted Wang-Colburn model of LSO. **A**: Circuit diagram of the adjusted Wang-Colburn model. **B**: Membrane impedance of the model. **C**: Current-potential (I-V) relationship of the model. **D**: Model responses to step current input with three varied sizes. **E**: Membrane responses to modeled excitatory and inhibitory synaptic inputs. **F**: (Left) Modeled membrane potentials driven by binaural AM tones with two different input phase differences. (Right) Output rates of the model in response to binaural AM tones with varied input phase differences. Bold numbers show the peak and trough rates. **G**: (Left) Modeled membrane potential driven by binaural unmodulated tones with two different ILDs. (Right) Output rates of the model in response to binaural unmodulated tones with varied ILDs. Bold numbers show the rates at -45 dB and +15 dB. In panels F (Left) and G (Left), horizontal dotted gray lines indicate the resting potential. **H**: Monaural AM-tuning curve (rate-MTF) of the adjusted Wang-Colburn model. Bold numbers show the peak rate and the rate at 1200 Hz. (Inset) Monaural phase-locking (synch-MTF) of the model. Blue rectangular shading in F-H indicates the targeted ranges, while green shading in F-G shows the accepted ranges. **I**: Binaural AM phase-tuning curves of the model at three modulation frequencies. **J**: Binaural ILD-tuning curves of the model at five ipsilateral sound levels.

The adjusted Wang-Colburn model has the same type of ion channels (Fig 10A) as the original version with a voltage shift of +5 mV. The membrane capacitance and resistance were reduced to fit corresponding data [77]. The membrane impedance profile (Fig 10B) and I-V curve (Fig 10C) resembled those for the active IF model. The adjusted model showed tonic (repetitive) spiking for a lower step current input (Fig 10D, right) than the non-adjusted model. Shapes of inhibitory inputs (Fig 10E, bottom) better resembled the *in vitro* data [77]. The model still shows large, overshooting spikes (Fig 10E, top), as the reversal potential of the sodium conductance was unchanged from the original version (see Discussion). With these revisions, subthreshold traces were not greatly altered (compare traces in Fig 10F and 10G with traces in Fig 9F and 9G), but the binaural tuning curves yielded a larger modulation depth in phase coding (Fig 10F, right) and a higher peak for level coding (Fig 10G, right).

Simulated monaural AM tuning curve (Fig 10H), binaural phase-tuning curves at different modulation frequencies (Fig 10I), and binaural level tuning curves with varied ipsilateral levels (Fig 10J) all resembled empirical data (Fig 1B–1D), although there were still some minor discrepancies. The monaural spike rate at 1.2 kHz (Fig 10H), for example, was higher than most units in cat LSO (Fig 1B). The peak of the monaural tuning curve (Fig 10H) was steeper than other models. Furthermore, the peak height for the binaural level tuning curves had a ‘ceiling’ at about 130 spikes/s (Fig 10J), because the outward rectification and Na inactivation still had considerable effects in the adjusted model.

### Comparison of credibility and computational efficiency

**Fig 11:**
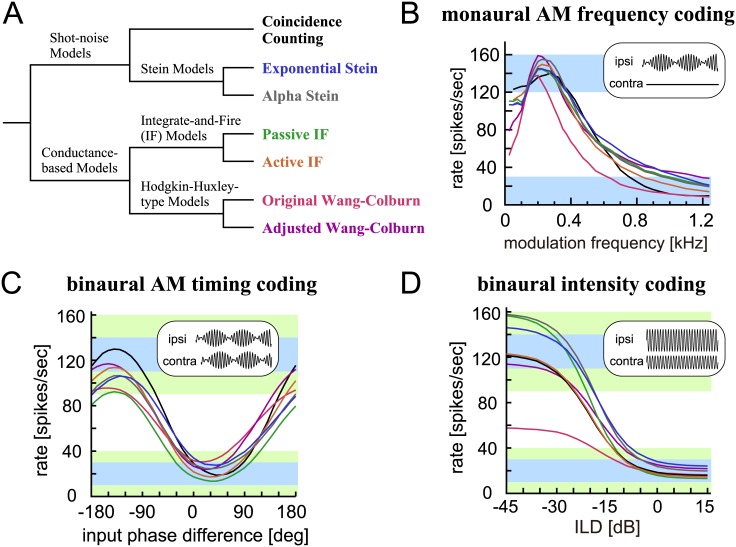
Summary of simulated tuning curves of the models. **A**: Interrelations of the seven LSO models used in this study. **B**: Monaural AM-tuning curves (rate-MTFs) of the models. **C**: Binaural AM phase-tuning curves of the models at the modulation frequency of 300 Hz. **D**: Binaural ILD-tuning curves of the models at the ipsilateral sound level of 35 dB. Line colors in B-D correspond to the text color in A. Blue rectangular shadings indicate the targeted ranges, while green shading show the accepted ranges.

In previous sections, we have characterized the response properties of seven LSO neuron models. Mutual relations of the models are shown in Fig 11A. Monaural and binaural tuning properties of the models are summarized in Table 1. We used monaural AM tuning, binaural phase tuning, and binaural intensity tuning curves for selecting the parameters. In order to quantify model performance for comparison, we set a criterion for the peak, trough, and depth of these three curves, yielding nine targeted ranges in total (see Materials and methods for their definitions). All models satisfied the target ranges for monaural AM tuning showing similar band-pass curve shapes with a peak at around 200–300 Hz (Fig 11B). Binaural phase tuning curves of the models were also similar to each other with some variations in modulation depths (Fig 11C), while their binaural intensity tuning curves considerably differed particularly in peak amplitudes (Fig 11D; see also Discussion). The coincidence counting model, the active IF model and the adjusted Wang-Colburn model satisfied all the targeted ranges (as shown by the bold numbers in Table 1). For the other models, we weakened the criteria by introducing wider accepted ranges. All models except the original Wang-Colburn model achieved the accepted ranges for the nine output rates (non-bold numbers in Table 1).

**Table 1:** Summary of model output measures.

Model	Monaural AM frequency coding			Binaural AM phase coding (at 300 Hz)			Binaural intensity coding (at ipsilateral level of 35 dB)			Relative computational time
	Peak	Trough	Depth	Peak	Trough	Depth	Peak	Trough	Depth	
Coincidence counting (**9**–9)	**140.2**	**9.5**	**130.7**	**129.9**	**18.8**	**111.1**	**121.5**	**15.8**	**105.7**	1.0
Exponential Stein (**6**–9)	**145.1**	**22.5**	**122.6**	106.1	**27.5**	78.6	146.0	**24.1**	**121.9**	1.9
Alpha Stein (**6**–9)	**155.0**	**20.5**	**134.5**	106.5	**24.4**	82.1	157.7	**19.7**	**138.0**	3.0
Passive IF (**6**–9)	**144.3**	**21.4**	**122.9**	92.3	**13.5**	78.8	156.6	**13.1**	**143.5**	6.3
Active IF (**9**–9)	**149.6**	**15.0**	**134.6**	**113.8**	**17.3**	**96.5**	**123.0**	**14.7**	**108.3**	29.4
Original Wang-Colburn (**4**–6)	**137.9**	**9.0**	**128.9**	95.6	30.6	*65*.*0*	*57*.*6*	**16.4**	*41*.*2*	137
Adjusted Wang-Colburn (**9**–9)	**158.9**	**29.0**	**129.9**	**117.0**	**23.8**	**93.2**	**113.9**	**21.8**	**92.1**	137
Accepted ranges (spikes/sec)	100–180	0–50	>90	90–160	0–40	>70	90–160	0–40	>70	---
**Targeted ranges** (spikes/sec)	**120–160**	**0–30**	**>110**	**110–140**	**10–30**	**>90**	**110–140**	**10–30**	**>90**	---

The unit of the spike rates (column 2–10) is spikes/sec. (Peak): peak rate; (Trough): trough rate; (Depth): modulation depth. Simulated spike rates that were within the targeted range are shown in **bold** with blue background, while those out of the targeted range but still within the accepted ranges are shown with regular fonts with green background. Simulated rates that were out of the accepted ranges are shown as *italic* with red background. The numbers in the brackets after each model name show how many of the nine **targeted**-accepted ranges were attained. See Materials and methods for the definition of each model and output measures.

In addition to the credibility of the model, which is characterized by how well the model reproduces corresponding empirical data, computational performance is another important factor to evaluate a model. Relative computational time increased with the complexity of the model (Table 1, rightmost column, from top to bottom). The coincidence counting model was more than 100 times faster than the Wang-Colburn model, followed by the Stein models (40–70 times faster) and the passive IF model (about 20 times faster). Calculation of the nonlinear dynamics of KLVA conductance considerably reduced the speed for the active IF model, because the model became a system of two differential equations, making it more than 4 times slower than the passive model. Additional nonlinear conductances made the computation even slower by another factor of over 4 in the Wang-Colburn models. It should be noted that we used the simple forward Euler method with a fixed time step to measure the relative computational time. Selection of a proper integration method in combination with an adaptive time step may reduce the computational time without losing the computational accuracy [89]. Therefore the relative computational time shown here should be regarded as one possible measure for evaluating the computational performances of the models.

In summary, the coincidence counting model yielded better fit to the targeted ranges with less computational costs than the Stein models and the passive IF model, whereas the active IF and adjusted HH model required more computational time to make similarly good physiological predictions. In Discussion, we re-examine the physiological and computational performances of each model, and provide our suggestions on possible applications of these models.

## Discussion

### Comparing models

Having multiple working hypotheses has long been suggested to be advantageous in scientific studies over sticking to only one (possibly flawed) ‘ruling’ hypothesis, because comparison of hypotheses is more likely to reveal various causes of a complex phenomenon by encompassing it from several sides [90–92]. Comparative research across animal species, for example, allows us to identify general functions of the subjects under study [93,94]. Similarly, comparison of different models helps us reveal which specific assumption results in what outcome, providing insights about the common operating principles of the system. In this study, we examined seven physiological models of LSO, which simulated neuronal processing of monaural and binaural acoustic information relevant to sound localization. The outcome of each model was compared with available empirical data as well as with simulation results of other models.

#### Comparison of model with empirical knowledge

Discrepancies between model outcome and empirical observations indicate that some of the original model assumptions are inappropriate and suggest what additional factors need to be considered in a revised model [4,5]. In our LSO modeling framework, the structure and most of the parameters of each model were determined from corresponding empirical data that were taken in prior physiological (mostly *in vitro*) studies. Parameters for which no experimental data were available were subject to fitting, in which simulation outcome was compared against known spiking output of LSO neurons recorded *in vivo*. For example, the original Wang-Colburn model was capable of reproducing a monaural AM tuning curve (Fig 9H) but not a binaural level tuning curve (Fig 9J). However, by adjusting the model parameters and ion channel kinetics, an improved fit to empirical results was achieved (Fig 10H–10J). To test whether the adjustments of ionic conductances and channel kinetics are supported by empirical observations will be a subject of future studies.

The plausibility of a model is generally judged by the agreement between its underlying assumptions and corresponding empirical knowledge, but biological realism does not always equate with a good predictive performance. Models that were proven to be constructed on wrong or inappropriate assumptions often produce good (or sometimes excellent) predictions [65], which may be attributed to "trade-offs among model components" [2]. Many auditory periphery models, for example, (incorrectly) assume that different frequency channels operate independently of each other, but still generate more than reasonable results [67]. The spike generation and succeeding potential reset of the IF model is far from empirical reality, but nevertheless the IF model and its variations have served as an important means to study the spiking behaviors of a wide variety of neurons [9]. In our modeling framework, the coincidence counting model naively counts the number of incoming synaptic inputs for a fixed duration of time with equal weights and discards all other inputs (Fig 4A and 4B). This assumption is biologically unrealistic, but the model still predicts both monaural and binaural properties well (Fig 4E–4G).

#### Comparison between models

In the study of complex systems, an increased confidence on the underlying, universal mechanisms is achieved when multiple models yield similar or identical predictions, whereas differences in model predictions implicate the necessity of further investigations to falsify some of the model assumptions. Such comparisons were performed, for example, with models of visual cortical maps [95], with myelinated auditory nerve fiber models [96], with basilar membrane models of the cochlea [67], and with compartmental models of neocortical pyramidal neurons [66]. A comparison of the leaky IF model with exponential IF and HH-type models suggested that interspike intervals driven by fluctuating inputs can be described by a gamma distribution, independently of detailed spike generation mechanisms [97], whereas a variation of IF model was shown to reproduce spike initiation of cortical neurons more realistically than detailed HH-type models [98].

Among our LSO models, the lack of compressive factor in the passive (linear) models led to a higher output rate when driven by high intensity inputs (Figs 6G and 7J) than in models with low voltage activated currents that lowers the membrane impedance at high membrane potentials and thereby reduces the effect of summed synaptic inputs (Figs 8J and 10J). This comparison (as shown in Fig 11D) suggests that the nonlinear suppression may stabilize the ILD processing of LSO by restricting the range of output spike rates. A prior study using a KLVA channel model showed that the dynamic properties of KLVA current are indeed important for auditory coincidence detection [99]. These results demonstrate that models with biophysical details (such as HH-type) can be useful in identifying important mechanisms that cannot be captured in simplified models.

The limit frequencies of phase-locking to monaural AM sounds differed between models: synch-MTFs of the HH-type models decayed at lower modulation frequencies (Figs 9H and 10H, inset) than those of other simpler models (Figs 5E, 6E, 7E and 8E, inset), even though the rate modulation transfer functions were similarly tuned. Future experimental studies may prove which model predictions on synch-MTFs are more realistic, whereas further theoretical investigations are needed to reveal the underlying mechanisms causing these discrepancies.

### Physiological models of LSO

In most earlier modeling studies, the function of an LSO neuron was abstracted as an interaction of excitatory and inhibitory inputs that determines the output spike rate [41–44,46]. In contrast, as empirical data accumulated, more physiological modeling approaches became prevalent [45,47–53]. These models were constructed on available *in vivo* and *in vitro* recording data: e.g., the ionic conductances and time constant of the membrane, synaptic time scales, spiking responses to monaural and binaural sound stimuli, and refractoriness. Such a physiological model is sometimes called a ‘pinkbox’ model [54], making a contrast to functional, ‘blackbox’ models that solely focus on the input-output statistics of a neuron [9]. The credibility of a physiological model is warranted by the solid connection between its underlying biophysical processes and resulting spiking behavior.

Within the category of physiological models, several levels of description may exist, resulting in a number of different models explaining the same phenomena. In the present study, we presented the models in order of ascending complexity, from the most simplistic coincidence counting model (Fig 4) to the elaborate HH-type models (Figs 9 and 10), having Stein and IF models (Figs 5–8) in between. Previous theoretical studies connected several levels of description by using various techniques in reducing models: e g., the reduction of a HH model into a threshold model [100], into a two-variable model [101] or into a variation of active IF models [102–104], and the reduction of a spike neuron model into a rate model [105]. Our active IF model of LSO is closely related to an MSO model with KLVA conductance and thresholding [106], which was introduced as a reduced description of more-detailed conductance-based model.

#### Simple vs. complex models

The balance between simplicity and reality is always a crucial issue in modeling studies (e.g., [5,7,9,10,107]). A biophysical model needs to be detailed enough to assure biological fidelity (and generalizability), while it should be sufficiently simple (or abstract) to allow for theoretical analyses or interpretations. This trade-off is also described as the philosophical debate between reductionism and holism [2]: a reductionist approach, frequently seen in applied mathematics and physics, aims at decomposing the system into parts to identify essential features, while a holist approach tries to include (almost) every component to replicate the emerging integrative behaviour of the complex system. These two philosophies, however, are not mutually exclusive, but complement each other to advance our scientific knowledge [7]. The simplistic coincidence counting model highlights the LSO neuron as a coincidence detector [40], while the detailed Wang-Colburn model describes how each biophysical element may play a role in temporal processing of auditory signals [53], together revealing the computational principles of LSO from different aspects.

In constructing a model, a general rule-of-thumb is that the complexity of a model should not exceed the complexity of the question that the model is addressing [8]. And according to the "principle of parsimony" (e.g., [1,92]), simpler models are favored over complex models if they have the same predictive capabilities, because simple models are more transparent and easier to communicate, and usually require less effort for parameter optimizations and model validation [2]. The meaning of each parameter of a simple model, however, tends to be more abstract and thus harder to relate with its underlying processes. The coincidence window in the coincidence counting model, for example, is determined by complex interactions of several biophysical factors, such as the membrane and synaptic time constants and nonlinear activation kinetics of ion channels [40]. The contribution of each of these factors cannot be easily evaluated separately from each other. Furthermore, it is difficult to directly incorporate measured nonlinear conductances into the shot-noise models, since these models lack detailed membrane dynamics.

In theoretical studies, finding the minimal description of a system is important for revealing its essential components [8,107]. The coincidence counting model, which simply counts the number of synchronized inputs and generates a spike, is (close to being) minimal. Several earlier studies mathematically formulated the spike statistics of the exponential Stein model driven by random synaptic inputs [75,76,108,109]. The passive IF model has been widely used in various fields of theoretical and mathematical neuroscience, including analyses of neurons that receive phase-locked inputs [69,70].

Simple models, however, are usually less flexible than detailed models. In the coincidence counting model, for example, the spike count is always an integer, and therefore any adaptive mechanisms that require a finer tuning of the threshold cannot be represented by this model. Furthermore, the simple shot-noise models cannot be expanded with additional ion channels. Related to this, the Stein models and the passive IF model are incompatible with subthreshold nonlinearity, resulting in higher spike rates than more detailed models when driven by high intensity synaptic inputs (Fig 11D). Detailed conductance based models are required, for example, to investigate possible effects of neuronal morphology, ion channel distribution, and locations of synaptic terminals on resulting binaural computation (e.g., LSO: [45]; MSO: [99]).

In light of applications, ‘medium-sized’ models are practically useful, because "they are complex enough to have nontrivial and nonobvious results, but simple enough so that the implications of the assumptions are readily apparent" [110]. Such an intermediate model should retain essential features of more detailed models to gain important insights into the modeled system [111]. Our active IF model (Fig 8) is an example of such medium-sized models, which ignores complex spike-generation mechanisms but still faithfully reproduces known sub- and suprathreshold characteristics of LSO neurons. In order to reduce the computational costs of the active IF model, the spike-associated current can be replaced by a simple potential rest and a succeeding absolutely refractory period (as in the passive IF model), when the entire spike waveform is not important.

#### Which model to choose?

As already seen in Introduction, no model is perfect. Therefore the question that a model user should ask is not "What is the best model?" but "What is the special value of each model, and what is its advantageous applicability?" (see [90] for a similar statement on selecting scientific methods). Table 2 summarizes major strengths and weakness of the seven LSO models, which have been generalized and validated with the common set of criteria. Here we note our suggested use of these models: in order to simulate the input-output relationship of an LSO neuron without considering its biophysical details such as membrane potential, we recommend the coincidence counting model; its computational efficiency also suits large-scale simulations that involve thousands of neurons or engineering applications that require real-time computation. To theoretically formulate the function of a LSO neuron, shot noise models (coincidence counting and Stein models) or the passive IF model would be suitable, as there are number of relevant studies and techniques available. In order to study how specific membrane properties and their interactions (e.g., ion channel kinetics and conductance densities) may play a role in the excitatory-inhibitory interaction of an LSO neuron, the adjusted Wang-Colburn model is clearly advantageous over other simpler models, because of its well-founded biophysical details and potential expandability with additional ion channels and neuronal morphology. For a general purpose, or when the user does not have a strong preference on which model to choose, the active IF model would be a good compromise among biological reality, computational efficiency, and predictive credibility.

**Table 2:** Summary of model characteristics.

Model	Notes and recommendations
Coincidence counting	Extremely fast. Good predictions. Least flexible. Limited to modulation frequencies below ~1 kHz. Recommended for simulations where membrane potentials (and adaptation) do not really matter. May also be suitable for large-scale and/or real-time simulations.
Exponential Stein	Very fast. Relatively good predictions. With some flexibility. Possibly suitable for theoretical analyses. Not strongly recommended for explaining empirical data, as the low threshold and the shape of inputs may be biologically unrealistic.
Alpha Stein	Fast. Better biological plausibility than the exponential Stein model and with similarly good predictions. Possibly suitable for theoretical analyses. Due to the lack of subthreshold nonlinearity, recommended only with low-to-medium input intensities.
Passive IF	Relatively fast. With good biological bases and flexibility including the membrane potential. Similar predictive performance to Stein models. A number of analysis techniques are already available. Recommended only with low-to-medium input intensities.
Active IF	Relatively slow. With solid biological plausibility including the subthreshold nonlinearity. Better predictions than most other models. Expandable with additional (subthreshold) conductances. Recommended for general use, especially when subthreshold membrane potentials and/or spikes matter.
Original Wang-Colburn	Slow. With established biological background (based on recordings from VCN neurons), but with poor predictive performances. Not strongly recommended for simulating binaural responses (especially ILDs). Parameters clearly need revisions.
Adjusted Wang-Colburn	Slow. Good predictions. With solid biological bases, but with many unknown (experimentally unconstrained) parameters. May need further optimizations. Expandable with additional conductances. Suitable for studying how the kinetics of each ion channel may play a role.

### Limitations of the modeling framework

#### Parameter selection

For a systematic searching of model parameters, a number of computerized methods have been invented [66,112–115]. Selecting the parameters of a nonlinear dynamical system, however, requires attention to various types of potential pitfalls. A small change of a parameter may cause dramatic changes in the resulting behavior of the model, referred to as a bifurcation (e.g., [116]). Conversely, different parameter sets sometimes lead to practically indistinguishable results [113,117,118]. This is because the model can be highly sensitive to parameter changes in certain directions and not to those in other directions, and because changes in some model components can compensate each other [119] (see [120] for a review of these issues). Furthermore, averaging ‘good’ parameter combinations occasionally yield poor results [121], since the distribution of good parameters can be highly skewed in the parameter space (i.e., mathematically speaking, the distribution is non-convex). As the number of unconstrained parameter increases, the volume of the parameter space to search rapidly increases, making the parameter search inefficient, which is so-called the ‘curse of dimensionality’ [8,66]. In addition, since it is impossible to test a model completely with an infinite amount of empirical data, the modeler needs to define the desired range of applicability and to leave the model for further tests in domains where it was not optimized for [2,66].

For our conductance-based LSO models (i.e., the passive and active IF models and the adjusted Wang-Colburn model), we adopted a multi-step procedure for parameter tuning (as suggested, for example, in [122]) instead of using a fully automated parameter optimization technique, because corresponding empirical data were sparse and taken from various independent (and qualitatively different) sources. First, we focused on the subthreshold (non-spiking) responses of the neuron and tuned corresponding parameters (i.e., membrane conductances, reversal potentials, synaptic time constants and amplitudes, etc.). We then selected the parameters for suprathreshold spiking responses (e.g., thresholds of the IF models, and Na and KHVA conductances of the adjusted Wang-Colburn model) driven by simulated synaptic inputs, to meet the output criteria for monaural and binaural tuning curves, which we considered the most important response characteristics of the LSO neuron. After parameter selection, all models except the original Wang-Colburn model satisfied the nine output criteria (i.e., within all the acceptable ranges shown in Table 1). It should be noted, however, that we did not try to find the ‘best’ parameter combination for each model. In our preliminary simulations, multiple parameter combinations resulted in virtually identical response properties for the reasons discussed above. The model parameters we adopted in this study should thus be regarded just as a reasonable option (see Materials and methods for more details about our parameter selection).

#### Availability of empirical data

In this study, we selected the parameters of each model so that it replicates the three major response features of monaural AM tuning, and binaural phase and intensity tunings (Fig 11B–11D). For conductance-based models, sub- and suprathreshold responses were additionally used to calibrate their membrane properties. We focused on these three response features for two reasons: First, considering the binaural nature of this nucleus [12] and its behavioral relevance [123], we presumed that ITD and ILD tunings are the most prominent characteristics of LSO, and that monaural AM stimulus should be used to further characterize its responses to temporally structured excitatory inputs. Second, currently there is only limited information available that can be used for constructing and validating an LSO model. Except for the stimulus characteristics we adopted in this study, we practically do not have sufficient amount of quantitative data taken from LSO and its primary input sources (VCN and MNTB). It should also be noted that, in none of previous LSO experiments, were the three response features altogether tested in the same single neuron. Therefore, our criteria of simultaneously sufficing the nine targeted ranges (Table 1) might be too strict for a single neuron model. As more empirical data become available in future measurements, the models should be re-evaluated with a wider variety of stimuli (see also "Possible applications" below).

Earlier studies presented a ‘chopping’ behavior in the peristimulus time histogram (PSTH) to be a distinctive response feature of LSO [30,41,124], guiding subsequent modeling studies to replicate these activity patterns (e.g., [43,51]). However, we did not use PSTHs to tune or evaluate LSO models, because the observed chopping patterns were found to be influenced by anesthesia [125,126] and may further affected by time-delayed ipsilateral inhibition [126], which is beyond the scope of our present study.

#### Difference across species

In order to sufficiently constrain the LSO models, we had to borrow empirical data from various experimental studies. Most *in vivo* data that we used to measure the output performances of the models had been obtained in cats, while most *in vitro* data that we adopted to tune the subthreshold membrane properties had been taken from rodents. This type of problem has been "well known among old-timers" [127], and is still common when a modeler tries to relate different levels of description: e.g., connecting *in vitro* slice recording data with *in vivo* network dynamics; or connecting neuronal activity with behavioral outcome. Moreover, cellular properties may be considerably different even between closely related species and sometimes incompatible with each other. Measured membrane resistances of LSO neurons of rodents, for example, were 42 ± 21 MΩ in gerbils [77] and 15–53 MΩ in mice [88], while they were much higher in guinea pigs (73 ± 17 MΩ: [48]) and in rats (109 ± 64 MΩ: [85]). Possibly related to this, action potentials in rats’ LSO neurons usually overshot with amplitudes of 81.3 ± 15.3 mV [85], whereas those in gerbils were substantially smaller (41 ± 14 mV: [77]). Furthermore, for technical reasons, *in vivo* recordings are performed normally with adult animals, while *in vitro* patch clamp recordings are done with juvenile animals whose cellular and synaptic properties may still be under development [128–130]. These discrepancies suggest that our models could represent an LSO neuron of a ‘chimera’ that may not exist on earth, although the predictions of the models were at least qualitatively consistent with known empirical data from real animals. Further experimental recordings and model optimizations would therefore be necessary to create an animal-specific LSO model.

#### Difference within species

Even within the same species, LSO neurons show considerable variations in monaural and binaural responses [37–39,124]. Previous physiological and anatomical studies of LSO found tonotopic gradients in the number of inhibitory synaptic receptors [131], the morphology of dendritic arbors [132], the KLVA conductance and resulting spiking patterns [86], and the resonance property of the membrane [48]. These tonotopic differences may explain some of the observed unit-to-unit variations among LSO cells. Our previous modeling study demonstrated that variations of the parameters for coincidence detection indeed affect the shape of the monaural and binaural tuning curves [40]. Nevertheless, earlier physiological studies of LSO, either *in vivo* or *in vitro*, presented their data often without the information of the estimated characteristic frequencies of the recorded units. Due to such lack of data, we did not take tonotopic variations into account when we tuned the models. Systematic recording, analysis and optimization will thus be needed for constructing frequency-specific LSO neuron models that can be used as a building block for future binaural network simulations involving across-frequency integration.

### Future directions

#### Possible extensions

In this study, we examined seven single-compartment LSO models with varied levels of complexity (Table 2). Our list, however, is far from exhaustive. Between the passive IF and HH-type models, for example, there would also be a number of nonlinear IF models, including the quadratic IF [133,134], exponential IF [135] and generalized IF models [136], which we expect to show similar response properties to our active IF model. In order to make the model biologically more plausible, various types of ion channels that were found in LSO and other auditory areas may be added to the Wang-Colburn model, such as persistent and resurgent Na^+^ channels [137], a number of different low- and high-voltage-activated K^+^ channels [138], and hyperpolarization-activated cation channels [139,140].

The spike initiation site of a principal neuron in the gerbil MSO is located in the axon at a distance of several tens of micrometers away from the cell body, where backpropagating, small spikes were observed [141]. Considering the morphological similarities between LSO and MSO neurons [13,132,142,143], remotely initiated action potentials could also explain non-overshooting spikes observed in whole-cell recordings of gerbil LSO neurons [77]. In order to simulate such backpropagating spikes, the model needs to have at least two compartments (e.g., [83,107]). Segregation of the synaptic integration site from the spike initiation site may also improve the biological plausibility of the model by reducing the (unrealistically) large sodium conductance of the cell body in the Wang-Colburn models (see [66] for related discussion with cortical neuron models). Furthermore, to incorporate the effects of dendritic integration of synaptic inputs mediated by active conductances, multicompartment models should be considered, as was done with other auditory coincidence detector neurons (e.g., [99,144]).

#### Possible applications

In this study we focused on the steady-state responses of LSO models driven by Poissonian input spikes. By replacing the input stage of the modeling framework with a more detailed model (e.g., [145,146]), responses to transient stimuli (such as click trains) or naturalistic sounds (such as vocalization and speech) may be simulated [48]. In such cases, the output stage (i.e., LSO model) might also have to be revised by incorporating an adaptive threshold, so that the model could more faithfully compute the temporal information of sounds [147,148]. These modifications would allow us to predict and examine how LSO neurons may respond to more natural stimuli that simultaneously contain both ITD and ILD information [36,37]. Further generalizability of the LSO models should be tested, when sufficient empirical data become available in future physiological measurements with more complex sound stimuli.

As discussed earlier, modeling enables us to simulate hypothetical situations that cannot be easily reached with experimental approaches. Predicting the effects of pathological changes in the auditory brainstem, for example, would be a subject of such modeling studies. Age-related loss of inhibitory neurons is commonly found in various animal species (see [149,150] for reviews); synaptic and membrane properties are also affected by aging [151]. Degraded localization performance for high-frequency sounds in aged animal models [152] and humans [153] suggests that altered inhibitory inputs from the MNTB may affect the function of LSO. A modeling approach with a varied number and amplitude of synaptic inputs would reveal how binaural phase- and level-tuning curves may be influenced by the loss of synaptic inputs, and provide fundamental insights relating prior anatomical [154] and physiological [155,156] findings in aged animals. For another example, potential effects of altered connectivity in a disease model mouse on binaural coding [157] could also be simulated by incorporating observed changes in the modeling framework.

Binaural information processing is critical for efficient acoustic communication both in animals and in humans, especially in a noisy environment [11,54]. How to preserve and use binaural cues in cochlear implant users has been actively investigated (see [158] for a review). Combining electric hearing models with binaural neuron models [58,59] would be useful not only to predict how bilateral implantation may or may not contribute to the improvement of perceptual outcome, but also to highlight future directions towards the refinement of coding technologies. Such an application would constitute an important step for a neuronal modeling study, since it provides an opportunity to make ‘out-of-domain’ predictions beyond the initial scope of the original modeling endeavor [122].

### Conclusions

In this study, we examined and compared the biological grounds, simulated responses, computational costs, and further expandability of seven neuronal models of the lateral superior olive (LSO). Envisioned applications of these models may range from basic research for investigating biophysical mechanisms of binaural information processing, to biomedical and engineering applications for better human and machine hearing. Based on our comparison results, we obtained the following conclusions:

- A wide variety of single-compartment LSO models can be calibrated to fit known basic physiological properties, including monaural AM coding, binaural phase and intensity coding.
- For applications where computational efficiency is required, the coincidence counting model is most suitable.
- For mathematical analyses to study the input-output relationship of LSO, shot noise models and/or the passive IF model are suggested.
- For a general purpose where no strong preference of models exists, the active IF model may serve as a useful starting point because of its balance between simplicity and biological plausibility.
- For simulations where biological details of ion channels kinetics, conductance densities, and morphological expandability are required, the HH-type Wang-Colburn model should be used.
- As more comprehensive empirical data sets become available in future experiments, the models will have to be recalibrated and revalidated.

## Materials and methods

### Common input

Our LSO modeling framework consists of two stages, input and output. In the input stage, sound stimuli are converted into simulated spike sequences of excitatory and inhibitory inputs to the model LSO neuron. In the output stage, the model neuron ‘processes’ these inputs according to its specific rules and produces output spikes. Since the main aim of this study was to compare various types of LSO neuron models, we fixed the input stage and evaluated the output of each model neuron. In this section, we first define the common input stage to be used with all LSO models. In next sections, evaluation criteria and detailed model descriptions are provided.

The major input sources to the LSO neuron are spherical bushy cells in the AVCN and principal neurons in the MNTB, which provide excitatory and inhibitory synaptic inputs, respectively (Fig 1A). In our modeling framework, simulated excitatory and inhibitory spike trains of these neurons were used as the common input to drive all LSO models. Other input sources are not considered, although they might modify the neuronal activity of LSO neurons [126]. We assumed that the activity of these AVCN and MNTB fibers (driven by modulated or unmodulated tones) can be described as an inhomogeneous Poisson process [78,79] with a time-varying intensity function λ(*t*). As in our previous study, we assumed that an LSO neuron receives 20 excitatory input and 8 inhibitory inputs (see [40] for the justification of these numbers). The activities of these input fibers were assumed to be statistically independent from each other.

Table 3 summarizes the equations we used for generating inputs to LSO models. Since the spiking activity of bushy cells and MNTB neurons are generally similar to each other (e.g., [34]), we assumed the same frequency and level dependence for both types of neurons. For AM tones, we considered the situation where the sound level was fixed and the modulation frequency and relative phase of the envelope between the two ears were varied. The intensity function λ(t) of input fibers are locked to the modulation frequency *f*_m_, with λ_1_(*f*_m_) being the frequency-dependent average intensity and *p*_*k*_(*x*) being a 2π-periodic function. We used a von-Mises distribution function [159] for *p*_*k*_(*x*), where the degree of phase-locking measured as vector strength (VS) [160] was parameterized by the concentration factor *k* (Table 3). For more detail about theoretical formulations, see [84]. As in our previous study [40], we adopted monotonically decreasing functions (Fig 2A and 2B) to roughly mimic empirical frequency dependence of intensity (λ_1_) and phase-locking (VS) of input fibers in gerbils [161] and cats [34,162].

**Table 3:** Equations and parameters for common input.

Variable / Parameter	Equation / Value
Periodic intensity function for inhomogeneous Poisson spike trains driven by AM tones	λ(*t*) = 2*π*λ_1_(*f*_*m*_)*p*_*k*_(2*πf*_*m*_*t*)
Modulation-frequency dependent average intensity	λ_1_(*f*_*m*_) = 180 − 0.03*f*_*m*_
von Mises distribution with concentration factor *k*	*p*_*k*_(*x*) = exp(*k* cos(*x*)) / (2*π*I_0_(*k*))
Modified Bessel function of order *n*	${\text{I}}_{n}(k)=\frac{1}{2\pi }\int_{-\pi }^{\pi }\text{exp}(k\;\text{cos}(x))\text{cos}(nx)dx$
Relations between VS and concentration factor	$\text{VS(}k\text{)}=\frac{{\text{I}}_{1}(k)}{{\text{I}}_{0}(k)}$
Frequency-dependent phase-locking (*f*_*m*_<2000)	$\text{VS}(f_{m})=0.65\;\times \frac{\left(1-\text{exp}(\;(f_{m}-2000)/500)\right)}{\left(1+\text{exp}(\;(f_{m}-2000)/500)\right)}$
Spontaneous activity	λ(*t*) = 30
Level dependent intensity function for Poisson spike trains driven by non-modulating tones	$\lambda (\text{SPL})=30+\frac{240}{\left(1+\text{exp}(-(\text{SPL}-20)/6.0)\right)}$
Number of excitatory input fibers	M_ex_ = 20
Number of inhibitory input fibers	M_inh_ = 8

*f*_*m*_ is the modulation frequency in Hz. SPL is the sound level in dB. The unit for the intensities λ and λ_1_ is spikes/s.

In our simulations, we first fixed the modulation frequency *f*_m_ and calculated the corresponding VS(*f*_m_); we then back-calculate the concentration factor *k* from the equation for VS(*k*); and we finally obtained Poissonian spike trains with this time-varying intensity function *p*_*k*_(*x*). We assumed that all input fibers (either excitatory or inhibitory) are locked to the same phase of the envelope when driven by AM tones. For ipsilateral monaural stimulation, the spontaneous activity of MNTB neurons was simply modeled as homogeneous (time-independent) Poisson trains without phase-locking.

To simulate ILD coding in the LSO (in response to non-modulated tones), we adopted a sigmoidal level-dependent intensity function λ(SPL) of input fibers (Table 3; Fig 2C). This equation roughly approximates known physiological recording results, which showed relatively large variations across species (cat VCN: [34,163]; monkey VCN: [164]; gerbil VCN: [165]; cat MNTB: [166,167]; rodent MNTB: [168]). We assumed that spiking activities of input fibers were not phase-locked for non-modulating tones, and hence the intensity function λ of the Poisson process used for ILD coding was intensity-dependent but time-independent.

### Parameter selection: Overview

The main goal of this study was to compare different types of LSO models with similar monaural and binaural tuning properties. Based on previous recording results (e.g., Fig 1), we determined a set of reference output, to which the parameters of our LSO models were tuned. For each model, tuning curves for monaural AM frequency coding (Fig 3A), binaural AM phase coding (Fig 3B), and binaural intensity coding (Fig 3C) were examined (see following subsections for their detailed descriptions). For each of these tuning curves, we set a ‘targeted range’ (bold numbers in Fig 3) and an ‘accepted range’ (non-bold numbers in Fig 3) of spike rates. This means that we have nine criteria in total for each optimized model to satisfy (Table 1). The performance of a model was measured by the number of targeted/accepted values achieved. We did not use the detailed shapes of simulated tuning curves as a primary measure of the performance, since no systematic data on corresponding curve shapes were available from previous experimental studies. For each parameter set, we calculated the average spike rates of the model neuron over 40 seconds.

It should be noted that multiple parameter combinations sometimes yielded virtually identical results, with the same number of targeted values attained (see Discussion). In such cases, we selected parameters that were closer to the corresponding empirical mean or median (if available), and had shorter digits (e.g., 1.2 rather than 1.2345). Care was also taken to ensure that a small variation (typically a few percent) in a single parameter value did not lead to a change in the hit/miss ratio of the target ranges, by avoiding parameter values with which simulated spike rates fell onto the ‘borderline’ of the targeted (or accepted) ranges. This means that the set of model parameters we used may not be the ‘only best’, but should rather be regarded as one of ‘reasonably good’ combinations of parameters that satisfy our target criteria (see also Discussion).

### Output measures

#### Monaural AM frequency coding

An LSO neuron changes its spike rate according to the modulation frequency *f*_m_ of ipsilateral AM tones (shown as an ‘**AM-tuning curve**’ in Fig 1B). This rate-*f*_*m*_ relation is also called the ‘**rate modulation transfer function’ (rate-MTF)** [169,170]. We calculated the rate-MTF of LSO models at modulation frequencies between 50 and 1200 Hz. Monaural AM coding can additionally be examined by the phase-locked output [34]. The ‘modulation gain’ was defined by 20log_10_(2*R*), where *R* is the VS of the LSO output spikes at the modulation frequency *f*_*m*_. This gain-*f*_*m*_ relation is also called the ‘**synchrony modulation transfer function’ (synch-MTF)** [170]. Since only insufficient empirical information is available, we did not use the synch-MTF for optimizing the model parameters, but used it as one of the output measures to characterize the models.

#### Binaural AM phase coding

When simulated binaurally, LSO neurons change their spike rates according to the interaural time (or phase) difference of the envelopes of the AM tones (shown as a ‘**phase-tuning curve**’ in Fig 1C). The interaural time difference is modified and compensated by several transduction factors, resulting in the time difference of bilateral synaptic input at each LSO neuron (e.g., [11,171]). We simulated this time difference at the LSO synapse by changing the relative phase of simulated synaptic inputs. As stated above, excitatory inputs were assumed to be locked to one particular phase of the envelope of the stimulus AM tone, while inhibitory inputs are locked to another phase. We varied the difference of these locking phases, and calculated the output spike rates of the models, first at the modulation frequency of 300 Hz (for parameter fitting) and then at 150 and 450 Hz (for further characterization). We defined a positive phase difference as inhibitory inputs preceding excitatory inputs as in previous experimental studies [36,37].

#### Binaural intensity coding

Because of the excitatory-inhibitory interaction, LSO neurons are generally sensitive to ILDs (shown as an ‘**ILD-tuning curve**’ in Fig 1D). Note that, by convention, the ILD is defined as the sound level at the contralateral ear minus that at the ipsilateral ear: i.e., a negative ILD means that the ipsilateral sound is louder. To simulate this ILD coding, we changed the binaural stimulus sound levels (of non-modulating sound) which drive excitatory and inhibitory inputs to the LSO model (Fig 2C). For parameter fitting, the ipsilateral level was first fixed at +35 dB and the contralateral level was varied between -10 and +50 dB, resulting in an ILD range between -45 and +15 dB. We then calculated the ILD-tuning curves with ipsilateral levels of +25, +30, +40, and +45 dB for further characterization of the model.

### Additional measures for conductance-based models

For conductance-based models (integrate-and-fire models and Wang-Colburn models: see subsequent sections for detailed descriptions), we used the following measures to tune the model parameters and evaluate their resulting membrane properties.

#### Membrane impedance

Input impedance of the model membrane was calculated by applying sinusoidal currents with a fixed amplitude of *I*_app_ = 10 pA and varied frequencies of 0.01–40 kHz. The membrane potential was clamped at -60 mV by an additional constant DC current. At each frequency *f*_app_, the maximum (*V*_max_) and minimum (*V*_min_) values of the steady state response (typically at >100 ms after the current onset) of the oscillating membrane potential were measured and the impedance was obtained as *R*(*f*_app_) = (*V*_max_-*V*_min_)/(2*I*_app_). In previous *in vitro* measurements in rats and guinea pigs [48], most LSO neurons had low-pass impedance profiles, while some other neurons that are found in the lateral (low-frequency) edges of the LSO showed weakly band-pass properties.

#### Current-voltage relationship

Current-voltage relationship **(I-V curve)** of the model membrane was obtained by applying step currents with varied amplitudes *I*_app_ between -0.5 and 1.5 nA. The membrane potential was clamped at *V*_clamp_ = -60 mV by an additional constant DC current. The spike generator (threshold-crossing detector) of the IF models and sodium channels of the Wang-Colburn models were disabled to avoid spiking responses. For each step current, the steady state response *V*_app_ (typically at >100 ms after the current onset) of the membrane potential was measured to plot an I-V curve. The DC input resistance of the membrane was calculated as *R*_DC_ = (*V*_app_-*V*_clamp_)/*I*_app._ with *I*_app_ = +10 pA.

In previous *in vitro* recordings, LSO neurons showed outward rectification [77,85]; i.e., the membrane resistance was lower at holding voltages above the resting potential than at or below. In the passive and active IF models and the adjusted Wang-Colburn model (see following sections for the definitions), we tuned the leak and other conductances so that the model membrane had an input resistance of 37–40 MΩ at the holding potential of -60 mV. This range corresponds to the measured values of 42 ± 21 MΩ in gerbils [77] and 15–53 MΩ in mice [88].

#### Step-current response

Step-current responses of the model membrane were obtained by applying rectangular currents with a fixed duration of 30 ms and varied amplitudes of 0 to 1 nA. The spike response was characterized as ‘**phasic**’ when the membrane stopped spiking before the offset of the step current, and as ‘**tonic**’ when the spiking activity lasted until the end of the step current.

Previous slice recordings in mice [88] and gerbils [77] reported that LSO neurons show tonic spiking with step currents of a few hundreds of pA; LSO neurons in rats showed a combination of tonic and phasic spiking responses depending on the size of the injected current [85]. This makes a contrast to other well-known auditory coincidence detectors of octopus cells in the posteroventral cochlear nucleus and principal neurons in the MSO, which have a large amount of low-threshold conductance and thus typically show only phasic spiking in response to step current injections [82].

### LSO models: Overview

In the following subsections, we provide the detailed descriptions of LSO models used in this study. All of them are single compartment models, which lack morphological structures (such as axons or dendrites) and thus receive synaptic inputs directly at the cell body (soma). Since the models have no internal noise sources, the model responses are deterministic; i.e., the model produces identical output for the fixed input. Trial-to-trial variability of the model output is solely due to the stochastic nature of simulated inputs.

The models can be categorized into either ‘**shot-noise models**’, in which synaptic inputs are directly reflected to the abstracted response of the model (virtual membrane potential), or ‘**conductance-based models**’, in which synaptic inputs and other ionic currents are described as temporally-varying conductances that eventually lead to the change in the modeled membrane potential (Fig 11A). The conductance-based models are further subdivided into two: ‘**integrate-and-fire (IF) models**’, in which spike generation process is abstracted as the detection of threshold crossing and succeeding reset of the membrane potential, and ‘**Hodgkin-Huxley (HH)-type models**’, in which sub- and suprathreshold responses of the membrane are fully described as the combined nonlinear dynamics of ionic conductances (Na^+^, K^+^, etc.). Of the seven models described below, the coincidence counting model, exponential Stein model, and alpha Stein model are shot-noise models; the passive and active IF models are (conductance-based) IF models; the original and adjusted Wang-Colburn models are HH-type models.

### Coincidence counting model

#### Model structure

The coincidence counting model of LSO is an extension of spike-count-based model of auditory coincidence detectors [172,173], and was fully described in [40]. In brief, the model compares the weighted numbers of excitatory and inhibitory inputs in a pre-set time window and generates an action potential when the total number reaches the threshold. More particularly, a coincidence window of size W_ex_ (Fig 4A, vertical gray band) slides along the time axis, and the number of incoming excitatory synaptic inputs (Fig 4A, blue bars) in this window is counted. If the number of input spikes in the window reached the pre-set threshold θ (Fig 4A, black bar), an output spike is generated (Fig 4A, green bar). If multiple threshold crossings happen within the pre-set refractory period T_ref_, then only the first one leads to an output spike and the others are discarded (Fig 4A, small arrow). When the model receives an inhibitory input, the threshold is elevated by a fixed amplitude H for the time length of W_inh_ (Fig 4A, dotted vertical rectangle). In other words, each inhibitory input subtracts the coincidence counts by H during the inhibitory time window of W_inh_. Note that the threshold θ is always an integer (i.e., non-integer values of θ do not make sense), as the model simply counts the number of synchronized inputs in the coincidence window.

#### Parameter selection and justification

The coincidence counting model has five free parameters (Table 4). The values we used were the same as the default values used in our previous study [40], which examined how each parameter affects the output of the model. In previous physiological experiments, measured refractory periods T_ref_ were 1.1–2.8 ms in cats [124]; measured length of inhibition window W_inh_ were 0.8–2.0 ms (rats) [174]; and measured thresholds θ were 9.6 ± 2.8 in gerbils [77]. In young gerbils [77], measured membrane time constants were 1.1 ± 0.4 ms and minimum durations of excitatory synaptic inputs were 1.5 ± 0.8 ms, both of which are expected to limit the maximum width W_ex_ of the coincidence window. Furthermore, based on the measured durations of excitatory and inhibitory inputs in gerbils [77], we assumed that the inhibition window W_inh_ was twice as long as the coincidence window W_ex_. Based on these experimental constraints, we determined the set of parameter values (Table 4) to satisfy the targeted criteria (Table 1) of output rates driven by simulated monaural and binaural inputs (Fig 4C–4E). Since there was no measurement available that showed how many excitatory inputs are cancelled by an inhibitory input, we choose the value of the inhibition amplitude H such that the binaural phase-tuning curve yielded a sufficient modulation depth (see Figs 11 and 12 in [40] for simulation results).

**Table 4:** Parameters for the coincidence counting model.

Parameter	Value
Refractory period T_ref_	1.6 ms
Coincidence threshold θ	8 inputs
Coincidence window W_ex_	0.8 ms
Inhibition amplitude H	2 inputs
Inhibition window W_inh_	1.6 ms

See text and [40] for justifications of the parameters and detailed examination of their effects.

### Exponential Stein model

#### Model structure

The exponential Stein model was adopted by Colburn and Moss [47] to study the excitatory-inhibitory interaction of LSO neurons (see [54] for a review of relevant earlier studies). In this model, each input gives rise to an exponentially decaying response of the virtual membrane potential (Table 5). The response is positive for excitatory inputs and negative for inhibitory inputs (Fig 5A); excitatory and inhibitory inputs generally have different amplitudes and decay time constants. When the potential reaches or exceeds the pre-set threshold θ, an output spike is generated. After each spike generation, the potential is reset and fixed to zero for the pre-set refractory period of T_ref_. Respecting the pioneering work by Stein [75,76], this type of model is called the ‘**Stein model**’ [109]. The term ‘exponential’ was added to clarify that each synaptic input is converted into an exponential function. This model was also called the ‘shot-noise model’ (e.g., [54]), but we leave this term for a more general category including the coincidence counting model (Fig 11A).

**Table 5:** Equations and parameters for the exponential Stein model.

**Variable**	**Equation**
Unitary excitatory input	J_ex_(*t*) = exp(−*t*/τ_ex_)(t ≥ 0), = 0(*t* < 0)
Unitary inhibitory input	J_inh_(*t*) = −H exp(−*t*/τ_inh_)(t ≥ 0), = 0(*t* < 0)
**Parameter**	**Value**
Refractory period T_ref_	1.6 ms
Threshold θ	5.5 inputs
Excitatory input time constant τ_ex_	0.70 ms
Inhibition amplitude H	1.8 inputs
Inhibitory input time constant τ_inh_	0.98 ms

Note that the excitation amplitude was fixed to 1.

#### Parameter selection and justification

The exponential Stein model has five free parameters (Table 5). As in other models of this study, we used the refractory period T_ref_ = 1.6 ms, which was comparable to the measured values of 1.1–2.8 ms in cats [124]. Other parameters were selected to fit the targeted monaural and binaural tuning curves. In our parameter selection, we initially explored the meshed four dimensional parameter space of θ (range: 6.8–12.4 inputs; step: 0.1), τ_ex_ (range: 0.5–2.0 ms; step 0.1), H (range: 1.0–3.0 inputs; step 0.1), and τ_inh_ (range: 1.0–3.0 times τ_ex_; step 0.2). The range of the model threshold θ was determined from the mean ± SD of measured threshold values in gerbils [77], whereas other parameter ranges were determined rather arbitrarily since there were no directly relevant data available for exponentially decaying synaptic inputs. In this parameter space, however, we did not find a set of parameters that satisfied all the nine accepted output criteria, primarily due to the low excitability of the model. Therefore we then expanded the range of the threshold as 5.4–13.8 inputs, which corresponded to the mean ± 1.5SD of measured values [77], and found parameter combinations (Table 5) that reproduced empirical monaural and binaural tuning curves (Fig 5C–5E). Although the shapes of inputs were considerably different between the model (exponential function) and *in vitro* slice recording data, the time scales of the modeled inputs (shown in Fig 5B) were roughly comparable to measured data (duration of excitatory inputs: 1.5–4.2 ms; duration of inhibitory inputs: 3.2–8.1 ms: [77]).

### Alpha Stein model

#### Model structure

The alpha Stein model is a direct modification of the exponential Stein model by replacing the exponential function with an alpha function (Table 6, Fig 6A), which is often used for modeling synaptic inputs [78–80]. In contrast to the exponential function that has a peak at the onset (i.e., at time zero) irrespectively to its decay time constant, the alpha function with a time constant τ reaches its peak at time τ. The model synaptic inputs (Fig 6B) showed similar shapes to empirical results [77,175]. Thus this modification makes the model biologically slightly more realistic at some additional computational costs (Tables 1 and 2). For other operations (such as the spike generation and refractoriness) of the alpha Stein model, the same rules apply as the exponential Stein model.

**Table 6:** Equations and parameters for the alpha Stein model.

**Variable**	**Equation**
Unitary excitatory input	J_ex_(*t*) = (*t*/τ_ex_) exp(1 − *t*/τ_ex_) (*t* ≥ 0), = 0(*t* < 0)
Unitary inhibitory input	J_inh_(*t*) = −H(*t*/τ_inh_) exp(1 − *t*/τ_inh_) (*t* ≥ 0), = 0 (*t* < 0)
**Parameter**	**Value**
Refractory period T_ref_	1.6 ms
Threshold θ	7.3 inputs
Excitatory input time constant τ_ex_	0.45 ms
Inhibition amplitude H	1.7 inputs
Inhibitory input time constant τ_inh_	0.63 ms

Note that the excitation amplitude was fixed to 1.

#### Parameter selection and justification

The alpha Stein model has five free parameters (Table 6). As in other models, the refractory period T_ref_ was fixed to 1.6 ms. We then determined the excitatory and inhibitory time constants (τ_ex_, τ_inh_) of the modeled synaptic inputs so that their time courses became similar to empirical data [77] and to simulated synaptic inputs in more detailed conductance-based models (compare Figs 6B and 7E). Finally we selected the threshold θ (range: 6.8–12.4 inputs; step: 0.1) and inhibitory amplitude H (range: 1.0–3.0 inputs; step 0.1) from the two dimensional parameter space, so that the model output satisfied the accepted output measures of monaural and binaural tunings (Fig 6C–6E).

### Synaptic inputs in conductance-based models

#### Model description

In the conductance-based (IF and Wang-Colburn) models presented below, alpha functions were commonly used to simulate synaptic conductances. Each synaptic input was converted into an alpha function (α_ex_, α_inh_), summed into the total conductance (g_ex_, g_in_), and then multiplied by the driving voltage to yield the resulting synaptic current (*I*_ex_, *I*_inh_) (Table 7). As noted before, modeled synaptic inputs are assumed to be injected directly into the soma.

**Table 7:** Equations and parameters for excitatory and inhibitory synaptic inputs commonly used with conductance-based models.

**Variable**	**Equation**
Unitary excitatory synaptic conductance	α_ex_(*t*) = A_ex_(*t*/τ_ex_) exp(1 − *t*/τ_ex_) (*t* ≥ 0), = 0 (*t* < 0)
Total excitatory synaptic conductance	${\text{g}}_{\text{ex}}(t)=\sum_{m=1}^{{\text{M}}_{\text{ex}}}\sum_{i=1}^{{\text{I}}_{\text{ex}}^{m}}\alpha_{\text{ex}}(t-t_{i}^{m})$
Excitatory synaptic current	*I*_ex_(t) = g_ex_(t) (E_ex_-*V*)
Unitary inhibitory synaptic conductance	α_inh_(*t*) = A_inh_(*t*/τ_inh_) exp(1 − *t*/τ_inh_) (*t* ≥ 0), = 0 (*t* < 0)
Total inhibitory synaptic conductance	${\text{g}}_{\text{inh}}(t)=\sum_{m=1}^{{\text{M}}_{\text{inh}}}\sum_{i=1}^{{\text{I}}_{\text{inh}}^{m}}\alpha_{\text{inh}}(t-t_{i}^{m})$
Inhibitory synaptic current	*I*_inh_(t) = g_inh_(t) (E_inh_-*V*)
**Parameter**	**Value**
Peak amplitude of excitatory input conductance A_ex_	3.5 nS
Peak amplitude of inhibitory input conductance A_inh_	12 nS
Time constant of excitatory input conductance τ_ex_	0.16 ms
Time constant of inhibitory input conductance τ_inh_	0.32 ms
Reversal potential for excitatory inputs E_ex_	0 mV
Reversal potential for inhibitory inputs E_inh_	-75 mV

*I*_ex_^*m*^ (*I*_inh_^*m*^) is the number of spikes of the *m*-th excitatory (inhibitory) fiber, *t*^*m*^_*i*_ is the timing of the *i*-th spike of the *m*-th fiber, and *V* denotes the model membrane potential. Note that the original Wang-Colburn model used different reversal potentials for synaptic inputs (see corresponding sections).

#### Parameter justification

The modeled excitatory E_ex_ and inhibitory E_inh_ reversal potentials were determined, respectively, from the standard driving voltage of AMPA receptors [78] and from the measurement of inhibitory postsynaptic potentials in gerbil LSO [77]. To calibrate the amplitudes and time constants, we used a simple RC membrane that has similar subthreshold electrical properties to gerbil LSO neurons [77]. The equation and parameters of the RC model was the same as the passive IF model (Table 8) but without any spiking thresholds. With the parameters shown in Table 7, simulated unitary amplitudes of excitatory and inhibitory postsynaptic potentials were about 2.3 mV and 2.7 mV, respectively, which were both within the measured ranges of 1.5–8.7 mV and 1.6–8.3 ms [77]. See Fig 7E for the shapes of modeled synaptic inputs.

**Table 8:** Equations and parameters for the passive IF model.

**Variable**	**Equation**
Membrane potential *V* (subthreshold dynamics)	$C\frac{\text{d}}{\text{dt}}V(t)=I_{\text{L}}+I_{\text{ex}}+I_{\text{inh}}+I_{\text{ext}}$
Potential reset after spiking	*V*(t_+_) → *V*_reset_ when V(t_-_) ≥ *V*_θ_
Leak current	*I*_L_ = g_L_ (E_L_-*V*)
External current	*I*_ext_ = 0 (default)
**Parameter**	**Value**
Membrane capacitance *C*	24 pF
Leak conductance g_L_	26.4 nS
Leak reversal potential E_L_	-60 mV
Reset potential *V*_reset_	-60 mV
Threshold *V*_θ_	-45.3 mV
Refractory period T_ref_	1.6 ms

Sanes [77] also measured the duration of synaptic inputs, which was defined as the time length "from the rising latency to the time at which the signal returned to the baseline noise level". Since our model lacks intrinsic noise sources, we calculated the difference of the two time points where the simulated unitary postsynaptic potential crossed the 5% line of the peak amplitude, and used it as a rough estimation of the duration. With the model parameters in Table 7, the simulated durations of excitatory and inhibitory synaptic inputs were about 3.5 ms and 4.1 ms, respectively. These values corresponded to the measured durations of 1.5–4.2 ms (excitatory) and 3.2–8.1 ms (inhibitory inputs) [77]. It should be noted that these measured amplitudes and durations of synaptic potentials showed considerable variations across LSO neurons, suggesting that the model parameter values we chose may not be optimal. In our preliminary simulations, however, we found that small variations in these input parameters (up to a few tens of percents) did not greatly alter the output of the LSO model, if other parameters were re-adjusted with the new input. Nevertheless, we also note that the duration of inhibitory synaptic potential needed to be around 3–5 ms or less, to fully account for the binaural phase-tuning curves.

### Passive IF model

#### Model structure

The passive integrate-and-fire (IF) model is the same as the standard leaky IF model used commonly in theoretical neuroscience [78–80]. We use the term ‘**passive**’ to make a contrast to the active IF model described below. The dynamics of the subthreshold membrane response is formulated as a linear (passive) RC circuit (Table 8, Fig 7A) with a low-pass impedance profile (Fig 7B) and a linear I-V relationship (Fig 7C). When the membrane potential *V*(*t*) reaches or exceeds the pre-set threshold *V*_θ_, an output spike is generated. After each spike generation, the membrane potential is fixed to the reset potential *V*_reset_ for a refractory period of T_ref_. It should be noted that, despite its name ‘integrate-and-fire’, an IF model with a short membrane time constant behaves rather like a coincidence detector than as an integrator of synaptic inputs [176].

#### Parameter selection and justification

The values of the six free parameters (Table 8) of the passive IF model were selected with a two-step procedure. First, experimental data were used to tune subthreshold membrane properties. As stated in the subsection titled "Current-voltage relationship", we determined the leak conductance g_L_ = 26.4 nS to obtain an input resistance of *R*_memb_ = 1/g_L_ = 37.9 MΩ. We then fixed the membrane capacitance *C* = 24 pF, based on previous physiological data [86], to obtain a membrane time constant of τ_memb_ = *R*_memb_*C* = 0.91 ms, which was comparable to the measured time constant of 1.1 ± 0.4 ms in gerbil slices [77]. The reversal potential E_L_ of the leak current was set at -60 mV, based on empirical resting potentials [77,85,86]. As the second step of parameter fitting, we searched for the value of the threshold *V*_θ_, so that the model output satisfied the criteria for monaural and binaural tunings (Table 1, Fig 7F–7H). The reset potential *V*_reset_ was the same as the resting potential E_L_. The length of the refractory period T_ref_ was the same as the shot-noise models, comparable to the measured value in cats [124].

### Active IF model

#### Model structure

The active integrate-and-fire model has a KLVA conductance (Fig 8A), which is the major source of the subthreshold nonlinearity of LSO neurons [86]. The term ‘**active**’ indicates the existence of voltage-dependent subthreshold currents. The model comprises two differential equations that describe the dynamics of the membrane potential *V*(t) and the KLVA activation variable *d*(t) (Table 9). Since the KLVA conductance increases at depolarized membrane potentials, the I-V curve shows an outward rectification (Fig 8C). As in the passive IF model, an output spike is initiated when the membrane potential reaches or exceeds the pre-set threshold *V*_θ_. After each spike (at time *t*_sp_), the model is in a refractory period of length T_ref_, during which the model generates no more spikes. In addition, a spike-associated current *I*_spike_(*t*-*t*_sp_) is injected to create a spike-like trajectory in the membrane potential to make the simulated responses somewhat more realistic than a simple potential reset [68].

**Table 9:** Equations and parameters for the active IF model.

**Variable**	**Equation**
Membrane potential *V* (subthreshold dynamics)	$C\frac{\text{d}}{\text{dt}}V(t)=I_{\text{L}}+I_{\text{KL}}+I_{\text{ex}}+I_{\text{inh}}+I_{\text{spike}}+I_{\text{ext}}$
Spike initiation	t^sp^ ≔ t when V(t) ≥ *V*_θ_
Leak current	*I*_L_ = g_L_ (E_L_-*V*)
KLVA current	*I*_KL_ = g_KL_ *d*(V) (E_K_-*V*)
Spike-associated current (*t* ≥0)	*I*_spike_(*t*) = 24 exp(−*t*/0.15) − 12 exp(−*t*/0.30)
External current	*I*_ext_ = 0 (default)
KLVA activation variable *d*(t)	$\frac{\text{d}}{\text{dt}}d(t)=\frac{d_{\infty }(V(t))-d(t)}{\tau_{d}(V(t))}$
Steady state for KLVA activation	$d_{\infty }(V)=\frac{\alpha_{d}(V)}{\alpha_{d}(V)+\beta_{d}(V)}$
Time constant for KLVA activation	$\tau_{d}(V)=\frac{1}{\alpha_{d}(V)+\beta_{d}(V)}$
Activation rate of KLVA	*α*_*d*_(*V*) = 0.5 exp(+(*V* + 50)/16)
Deactivation rate of KLVA	*β*_*d*_(*V*) = 0.5 exp(−(*V* + 50)/16)
**Parameter**	**Value**
Membrane capacitance C	24 pF
Leak conductance g_L_	14.4 nS
KLVA conductance g_KL_	21.6 nS
Leak reversal potential E_L_	-56 mV
Potassium reversal potential E_K_	-75 mV
Threshold *V*_θ_	-45.8 mV
Refractory period T_ref_	1.6 ms

Units: *t* is in ms, *V* in mV, *I*_spike_ in nA, and *α*_*d*_ and *β*_*d*_ in 1/ms.

#### Parameter selection and justification

The activation *α*_*d*_(*V*) and deactivation *β*_*d*_(*V*) rates determine the steady state activation *d*_∞_(*V*) and the time constant *τ*_d_(*V*) of the KLVA conductance (Table 9). Based on previous recordings from auditory neurons in various animals (mice: [177]; rats: [178]; guinea pigs: [179,180]; chickens: [181]), we defined the activation and deactivation rates using simple (and symmetric) exponential functions. As no data were available for the value of potassium reversal potential E_K_ of LSO neurons *in vivo*, we rather arbitrarily set it as -75 mV, which nevertheless corresponded to the values used in previous modeling studies of auditory neurons (e.g., -70 mV: [87,182]; -80 mV: [45]; -90 mV: [183]). The values of the leak g_L_ and maximum KLVA conductances g_KL_ were determined to reproduce empirical I-V curves [77], with a resulting input resistance of ~38.2 MΩ at the holding potential of -60 mV. The leak reversal potential E_L_ was determined so that the resulting resting membrane potential was between -61 and -60 mV.

After all the parameters for the subthreshold response were fixed, we selected the value of the threshold *V*_θ_ to attain the targeted rates of monaural and binaural tuning curves (Table 1, Fig 8F–8H). The shape of the spike-associated current *I*_spike_(*t*) was determined to mimic empirical spike shapes [77]. We used a sum of two exponential curves for *I*_spike_(*t*) (Table 9), because an exponential function enables exact calculations at discrete time steps [184]. The first and second terms of the spike-associated current are responsible for the depolarization and repolarization of the membrane potential, respectively. The length of the refractory period T_ref_ was the same as in the other models with thresholds.

### Original Wang-Colburn model

#### Model structure

Wang and Colburn [53] employed a conductance-based model to study the biophysical mechanisms of monaural frequency tuning of LSO neurons. Here we use the term ‘original’ to indicate that the model equations and parameters were unchanged from their original publication [53], except for the sodium reversal potential and the KLVA conductance (see below). The Wang-Colburn model has leak, KLVA, high-threshold-activated potassium (KHVA), and fast sodium (Na) conductances (Fig 9A). The kinetic equations for these conductances (Table 10) were directly taken from the Rothman-Manis model of guinea pigs’ VCN neurons [87]. The Wang-Colburn model is a variation of the HH model, and hence its spike threshold is not explicitly represented as a parameter but determined by a nonlinear interaction of ionic conductances. In our simulations, we counted a spike when the membrane potential depolarized above -30 mV and successively repolarized below -45 mV.

**Table 10:** Equations for the original and adjusted Wang-Colburn models.

Variable	Equation
Membrane potential *V* (subthreshold dynamics)	$C\frac{\text{d}}{\text{dt}}V(t)=I_{\text{L}}+I_{\text{KL}}+I_{\text{KH}}+I_{\text{Na}}+I_{\text{ex}}+I_{\text{inh}}+I_{\text{ext}}$
Leak current	*I*_L_ = g_L_ (E_L_-*V*)
KLVA current	*I*_KL_ = g_KL_ *w*^4^*z* (E_K_-*V*)
KHVA current	*I*_KH_ = g_KH_ (0.85 *n*^2^ + 0.15 *p*) (E_K_-*V*)
Fast Na current	*I*_Na_ = g_Na_ *m*^3^*h* (E_Na_-*V*)
External current	*I*_ext_ = 0 (default)
Kinetic equations for channel variables	$\frac{\text{d}}{\text{dt}}x(t)=\phi \frac{x_{\infty }(V(t))-x(t)}{\tau_{x}(V(t))}$ (*x* = *w*, *z*, *n*, *p*, *m*, or *h*)
Steady state for KLVA activation *w*	$w_{\infty }(V)={\left(\frac{1}{1+\text{exp}(-(V-V_{\text{shift}}+48)/6)}\right)}^{1/4}$
Time constant for KLVA activation *w*	$\tau_{z}(V)=1.5+\frac{100}{6\text{exp}(+(V-V_{\text{shift}}+60)/6)+16\text{exp}(-(V-V_{\text{shift}}+60)/45)}$
Steady state for KLVA inactivation *z*	$z_{\infty }(V)=0.5+\frac{0.5}{1+\text{exp}(+(V-V_{\text{shift}}+71)/10)}$
Time constant for KLVA inactivation *z*	$\tau_{z}(V)=50+\frac{1000}{\text{exp}(+(V-V_{\text{shift}}+60)/20)+\text{exp}(-(V-V_{\text{shift}}+60)/8)}$
Steady state for KHVA fast activation *n*	$n_{\infty }(V)={\left(\frac{1}{1+\text{exp}(-(V-V_{\text{shift}}+15)/5)}\right)}^{1/2}$
Time constant for KHVA fast activation *n*	$\tau_{n}(V)=0.7+\frac{100}{11\text{exp}(+(V-V_{\text{shift}}+60)/24)+21\text{exp}(-(V-V_{\text{shift}}+60)/23)}$
Steady state for KHVA slow activation *p*	$p_{\infty }(V)=\frac{1}{1+\text{exp}(-(V-V_{\text{shift}}+23)/6)}$
Time constant for KHVA slow activation *p*	$\tau_{p}(V)=5+\frac{100}{4\text{exp}(+(V-V_{\text{shift}}+60)/32)+5\text{exp}(-(V-V_{\text{shift}}+60)/22)}$
Steady state for Na activation *m*	$m_{\infty }(V)=\frac{1}{1+\text{exp}(-(V-V_{\text{shift}}+38)/7)}$
Time constant for Na activation *m*	$\tau_{m}(V)=0.04+\frac{10}{5\text{exp}(+(V-V_{\text{shift}}+60)/18)+36\text{exp}(-(V-V_{\text{shift}}+60)/25)}$
Steady state for Na inactivation *h*	$h_{\infty }(V)=\frac{1}{1+\text{exp}(+(V-V_{\text{shift}}+65)/6)}$
Time constant for Na inactivation *h*	$\tau_{h}(V)=0.6+\frac{100}{7\text{exp}(+(V-V_{\text{shift}}+60)/11)+10\text{exp}(-(V-V_{\text{shift}}+60)/25)}$
Voltage shift	*V*_shift_ = 0 mV (original Wang-Colburn model) *V*_shift_ = +5 mV (adjusted Wang-Colburn model)
Temperature correction	*$\phi ={\text{Q}}_{10}^{({\text{T}}_{\text{body}}-22)/10}$*

The kinetic equations are common for both models (from Wang and Colburn [53]; based on Rothman and Manis [87]), whereas the voltage shift was zero for the original model and +5 mV for the adjusted model. The temperature correction was common for all ionic conductances.

#### Adopted parameters

Wang and Colburn [53] tuned the parameters of their LSO model, focusing primarily on the monaural AM frequency coding (Fig 1B). The value of the maximum KLVA conductance g_KL_ was presented as "varied" in their report [53]. We adopted a value of g_KL_ = 85 nS, which was one of the values that had been reported to approximate empirical monaural tuning curves (Fig 9H). The sodium reversal potential E_Na_ was revised from 0 mV to +50 mV, because we found that the model with the old value failed to reproduce their reported results and later confirmed that the new value was indeed correct (Le Wang, personal communication, 2017). All other parameters shown in Table 11 are the same as the published values [53]. No further optimizations were performed with the original Wang-Colburn model.

**Table 11:** Parameters for the original Wang-Colburn model.

Parameter	Value
Membrane capacitance *C*	31.4 pF
Leak conductance g_L_	31.4 nS
KLVA conductance g_KL_	85.0 nS
KHVA conductance g_KH_	1200 nS
Na conductance g_Na_	8000 nS
Leak reversal potential E_L_	-65 mV
Potassium reversal potential E_K_	-70 mV
Sodium reversal potential E_Na_	+50 mV
Reversal potential for excitatory synaptic E_ex_	0 mV
Reversal potential for inhibitory synaptic input E_inh_	-70 mV
Temperature factor Q_10_	3.0
Body temperature T_body_	37°C

The temperature factor Q_10_ was common for all ion channels.

### Adjusted Wang-Colburn model

#### Model structure

Since the original Wang-Colburn model failed to reproduce binaural tuning curves (Fig 9F and 9G), we revised the model by shifting the voltage dependence of the channel kinetics and re-tuning the parameters. The term ‘adjusted’ indicates the revision from the original model. The model equations are the same as the original Wang-Colburn model (and thus the same as the Rothman-Manis model), except that an additional term *V*_shift_ was introduced to simultaneously adjust the voltage dependence of the sodium and potassium kinetics (Table 10). In our preliminary simulations, we found that, in the original Wang-Colburn model, activation of the large KLVA conductance in combination with the inactivation of the Na conductance prohibited repetitive spiking, resulting in low output rates (Fig 9J). In the adjusted model, we shifted the tuning curves of the ionic conductances by *V*_shift_ = +5 mV to reduce these effects. As noted above, we counted a spike when the simulated membrane potential exceeded -30 mV followed by an after-spike repolarization below -45 mV.

#### Parameter selection and justification

The sodium reversal potential E_Na_ and the temperature factor Q_10_ were the same as the original Wang-Colburn model (Table 12). For the membrane capacitance *C* and the potassium reversal potential E_K_, we adopted the corresponding values from the active IF model (Table 9). The synaptic reversal potentials E_ex_ and E_in_ were the same as the IF models (Table 7). The leak g_L_ and maximum KLVA conductances g_KL_ were determined to mimic empirical I-V curves [77] with a resulting input resistance of ~38.4 MΩ at -60 mV. Note that the outward rectification observed at or above -40 mV (Fig 10C) was also due to the large KHVA conductance. As in the active IF model, the leak reversal potential E_L_ was adjusted to obtain a resting membrane potential between -61 and -60 mV.

**Table 12:** Parameters for the adjusted Wang-Colburn model.

Parameter	Value
Membrane capacitance *C*	24 pF
Leak conductance g_L_	24 nS
KLVA conductance g_KL_	15 nS
KHVA conductance g_KH_	440 nS
Na conductance g_Na_	4400 nS
Leak reversal potential E_L_	-60 mV
Potassium reversal potential E_K_	-75 mV
Sodium reversal potential E_Na_	+50 mV
Temperature factor Q_10_	3.0
Body temperature T_body_	37°C

The temperature factor Q_10_ was common for all ion channels.

After fixing the above parameters, we selected the values of the sodium g_Na_ and KHVA conductances g_KH_ to satisfy our rate criteria of monaural and binaural tuning curves (Table 1, Fig 10F–10H). The enormous value of the Na conductance (compared to other conductances) was necessary to achieve a sufficient excitability of the model neuron, while the large value of KHVA conductance was required to ensure the repolarization after each action potential generation.

### Computational efficiency

We used the explicit (forward) Euler method for the numerical integration of the model equations. All simulations were performed with a time step 2 μs, although IF and shot-noise models generally allowed for longer time steps for stable and accurate calculations (see, e.g., [68]). In order to evaluate the computational cost of each model, we calculated the average integration time of twenty-five traces, each of which was 40-second long (i.e., 1000 seconds in total). To yield relative computational costs, we normalized the average integration time of each model by that of the coincidence counting model which required the shortest computation time. Numerical algorithms were implemented in D [185] and simulations were carried out on a desktop computer (Dell Precision T1700) with 64 bit Windows 7 Professional Operating System, Intel Xeon CPU E3-1270 v3 (4 core, 3.5 GHz) and a 16 GB memory. For readers’ convenience, Matlab implementation of the models is publicly available online at https://github.com/pinkbox-models.
